# Dietary rayon microfibers differentially reshape rearing water and host associated microbiomes of farmed European sea bass (*Dicentrarchus labrax*)

**DOI:** 10.1186/s40793-026-00851-5

**Published:** 2026-01-17

**Authors:** Fernando Naya-Català, Ricardo Domingo-Bretón, Ricardo S. Matias, Josep Àlvar Calduch-Giner, Álvaro Belenguer, Sónia Gomes, Lúcia Guilhermino, Federico Moroni, Luisa M. P. Valente, Jaume Pérez-Sánchez

**Affiliations:** 1 Fish Nutrigenomics and Integrative Biology Group, Instituto de Acuicultura Torre de la Sal (IATS) CSIC, Ribera de Cabanes, 12595 Castellón, Spain; 2 ICBAS, Instituto de Ciências Biomédicas, Abel Salazar, Universidade do Porto, Rua de Jorge Viterbo Ferreira 228, 4050-313 Porto, Portugal; 3 CIIMAR/CIMAR LA, Centro Interdisciplinar de Investigação Marinha e Ambiental, Universidade do Porto, Terminal de Cruzeiros do Porto de Leixões, Av. General Norton de Matos, S/N, 4450-208 Matosinhos, Portugal

**Keywords:** Marine pollution, Cellulosic microfibers, European sea bass, Gut microbiota, Skin microbiota, Water microbiota, Oxford nanopore sequencing

## Abstract

**Background:**

Viscose-rayon microfibres (RFs) are cellulosic microfibres widely dispersed throughout aquatic environments. Whether ingested by or suspended in the surrounding environment, these microfibres may impact both wild and farmed animals. A previous study on European sea bass (*Dicentrarchus labrax*) showed that the increased presence of RFs in aquafeeds (CTRL—no RFs; RF1—0.001 g/kg; RF2—0.01 g/kg; RF3—0.1 g/kg) was linked to an exponential increase of RFs in water, intestine and skeletal muscle. This finding was associated to a fatty liver and tissue-specific transcriptional changes, depicting the up-regulation of hepatic lipogenic enzymes and intestinal/head kidney inflammatory markers. The aim of the present study was to extend this evaluation by investigating changes in associated microbial communities after the ingestion of RFs in the diet, employing a multi-layered approach for the integrative profiling of gut, skin, and environmental water microbiome using the Nanopore platform.

**Results:**

Amplicon-sequencing identified ~2800 taxa across water, skin and gut microbiomes. Gut and skin microbiomes were more similar to each other, but increasing RF exposure shifted the skin community toward the water microbiome. Moreover, RF induced the highest taxonomic variation in water (691 taxa), followed by skin (253) and gut (99), while microbial diversity Shannon and Simpson indexes declined from 4 down to 3.3 under RF2 and RF3 in a dose-dependent manner. Major exponents of this trend were the decrease of *Synechococcus* and Flavobacteriales in association with the increase of starch- and hydrocarbon-degrading taxa (*Ardenticatenaceae* and Gracilibacteria). In both gut and skin, bacterial richness decreased in fish fed low to intermediate RF doses, whereas RF3 fish resembled controls. Thus, compositional and discriminant analyses consistently grouped CTRL and RF3 samples, suggesting the existence of a dose threshold occurring in parallel with host counter-regulatory responses. Such feature was reflected by abundant skin-associated bacteria (*Exiguobacterium *and *Planococcus*) with at least the genetic potential to be linked to vitamin B6 biosynthesis and host-driven muscle regeneration markers, whereas predominant gut taxa with the same pattern (*Microbacterium* and *Achromobacter*) was associated with polysaccharide degradation and correlated with host gene inflammatory mechanisms.

**Conclusions:**

This study revealed a concomitant dose-dependent and dose-threshold response among the bacterial communities composing the holobiont of European sea bass in response to dietary RFs ingestion, highlighting novel bacterial taxa and pathways through which microplastic exposure may differentially reshape rearing water and host-associated microbial communities.

**Supplementary Information:**

The online version contains supplementary material available at 10.1186/s40793-026-00851-5.

## Background

Microplastic (MP) contamination in aquatic environments poses a global threat to fish production systems, necessitating effective control measures to mitigate safety risks for living organisms and ecosystems [1–4]. When ingested, MPs can cause physical blockages, reduce nutrient absorption, and introduce toxic chemicals into fish tissues [5]. Upon sinking, MPs may also disrupt aquatic eukaryotic and prokaryotic biodiversity, undermining the health, stability, and sustainability of these environments [6]. Among MPs, increased attention is being directed to microfibres, tiny fibres of < 5 mm long and < 50 μm in diameter that constitute a major fraction of plastic micro-debris pollution [7]. Microfibers enter into both marine and freshwater ecosystems via sewage systems, which are estimated to discharge approximately 0.28 million metric tonnes (MMT) into the seas and oceans annually [8, 9]. This entry flow will endure over time, as textile manufacturing is ascending and expected to surpass 140 MMT by 2030 [10]. Such microfibres can be synthetic (i.e., petroleum-derivatives such as polyester, nylon, acrylic) or natural/semi-synthetic (cotton, wool, rayon), each one with distinct physical and chemical properties that affect how they behave in the environment and how organisms interact with them [11, 12]. Particular attention should be paid to non-synthetic fibres, which make up 67% of the mass of household dust and textiles [13]. These non-synthetic fibres are primarily cellulosic microfibres, which are also the most prevalent in the atmosphere, surpassing synthetic microfibres in a reported 57:23 ratio [14]. Likewise, recent studies have identified cellulosic microfibres as the predominant anthropogenic particles in both wild [15] and farmed fish [16]. Certainly, high levels of this type of microfibres have been detected in most aquafeeds, originated from anthropogenic action and aquafeeds ingredients, such as fish meal [17–19]. This seeks the importance of a precise risk assessment of these and other emerging pollutants to meet a more sustainable and ethical animal production in the aquatic environment [20–22].

In a previous study, Matias and co-workers [23] have investigated the physiological consequences of dietary exposure to increasing amounts of viscose-rayon microfibres (RFs) in European sea bass (*Dicentrarchus labrax*). These RFs are recognized as the most prevalent cellulosic microfibre in aquatic environments [24], and the results of the sea bass study highlighted that the increased dietary loads of RFs from zero to 0.1 g/kg led to their subsequent accumulation in the gastrointestinal tract, skeletal muscle, and surrounding water. Gastrointestinal levels (0.6–1.4 RFs/g) fall within the range of values reported for several Mediterranean organisms [25–27]. Muscle tissue concentrations (ranging up to 0.8 RFs/g) aligned with the upper limits reported for the coastal bottlenose dolphins (*Tursiops truncatus*) and Indo-Pacific king mackerel (*Scomberomorus guttatus*) [27, 28]. Likewise, seawater concentrations (0.13–0.40 RFs/L) associated to RF2 and RF3 diets slightly exceeded the MP levels previously reported in coastal Mediterranean waters [29]. Altogether, these findings indicate that the study by Matias and coworkers [23] established an experimental framework with exposure levels that largely reflect environmental conditions. Of note, no detrimental effects on growth performance or humoral factors were achieved over a 10-weeks trial with any of the tested RF doses. This observation mitigates the concern that natural changes occurring during host growth and development could act as confounding factors in our study [23]. Anyway, hepatic host transcriptional profiles revealed a shift towards lipid accumulation and some disruptions in bile acid metabolism, which were accompanied by compensatory remodelling mechanisms to preserve skeletal muscle growth and structure. Lastly, RF exposure induced a heightened pro-inflammatory state in the anterior intestine, coinciding with systemic immune suppression originating from the head kidney, which suggests a physiological trade-off that prioritizes localized gut immunity.

Beyond host physiology, attention needs to grow on the effects of RFs on the holobiont, the unity formed by the host and the microbial communities engaging interactions with it [30, 31]. Environmental pollution and ingestion of synthetic MPs (e.g., polyethylene or polystyrene) have been shown to strongly affect gut, skin, and water microbiomes, with important implications for fish health, resilience, and disease susceptibility [32–36]. The specific effects of cellulosic microfibres on host-associated microbiota remain less studied, even though recent work indicates that microbial populations in humans, livestock, and fish can respond to these fibres [37, 38]. Certainly, these microfibres may act as vectors for microbial pathogens, disrupting mucosal barriers, altering microbial composition, and influencing biofilm formation, with downstream effects on inflammation and immunity [39, 40]. In the skin, microfibres can provoke irritation and destabilize the cutaneous microbiota, thereby weakening host defence mechanisms [38, 41]. Waterborne microbial communities are also potentially affected, altering host-microbiota interactions and horizontal gene transfer [42]. In agreement with this, mussels and shrimp exposed to RFs showed a reduced microbial diversity along with increased abundance of opportunistic, xenobiotic-degrading, or virulent bacteria taxa [25].

Taking together all the above findings, a holobiont framework is thus essential to understand joint host–microbiota responses to anthropogenic pressures like RF contamination in aquatic organisms, and farmed fish in particular. Moreover, it is not clear if a specific RF dose is needed for microbial responses. However, integrated studies simultaneously evaluating the effects of RF exposure on host-associated microbiomes (gut, skin) and environmental microbiota (water) remain lacking to answer these questions. To address this gap, the use of Oxford Nanopore Technologies (ONT) protocols has recently been optimized for the simultaneous multi-compartment microbiome sequencing in European sea bass and other aquaculture-relevant species [43]. Using ONT tools, the main objective of the present study is to elucidate the effects of dietary RF exposure in the structure and inferred functions of intestinal, cutaneous and water surrounding microbial communities in European sea bass. At the same time, we also aimed to detect microbiome-specific effects and correlations with transcriptional and physiological responses of fish, as well as water microbiome variations with the presence of fish. Finally, we sought to determine whether microbial responses exhibit a dose-dependent pattern or a dose-threshold behaviour.

## Materials and methods

### Ethics statement

All the procedures within this trial were carried out in the Institute of Aquaculture Torre de la Sal (IATS), and approved by the IATS and CSIC Ethics Committee (permission 1135/2021), and Generalitat Valenciana (permission 2021-VSC-PEA-0192). Experiments aligned with the guidelines provided by the European Union Council (2010/63/EU) and Spanish legislation (Royal Decree RD53/2013) on the protection of animals used for scientific purposes.

### Experimental setup and sampling

European sea bass fingerlings of Mediterranean origin were obtained from a commercial fish hatchery (Avramar, Burriana, Spain). At their arrival, fish were acclimatized for 2 weeks in a 3,000 L tank, under a flow-through water system and were fed with a commercial feed (BioMar, Palencia, Spain). Then, 720 fish (5.8 g in average) were distributed in 12 tanks of 500 L, under a flow-through water system, with triplicate tanks per dietary treatment. Fish were fed extruded diets containing increasing levels of RFs (CTRL – no RFs; RF1–0.001 g/kg; RF2–0.01 g/kg; RF3–0.1 g/kg; 180 fish per diet). These dietary RF levels were chosen to be aligned with the previously reported range of microplastics and other anthropogenic particles in aquafeeds for European seabass (RF1), with the highest reported concentrations in aquafeeds (RF2), and with a microfibre concentration 10 times higher than any previously reported in aquafeeds (RF3) [23]. Diets were given to the fish during 68 days, three times per day (9:00 h, 12:00 h, and 14:30 h) with automatic feeders near to visual satiation. This was achieved by augmenting the feed supply to each tank by 2.5–3.5% daily, if no feed losses were observed. A complete water renewal was accomplished every 1.5 h, and water oxygen (O_2_) levels were monitored continuously, remaining above 75% air saturation (5–6.5.5 ppm) over the course of the trial. The daily water temperature ranged from 20 °C (May-2023) to 27 °C (July-2023), following the natural spring/summer variations at IATS latitude (40° 5’N, 0° 10’E). Fish used in the present study were from the same batch of those sourced in the work of Matias et al. [23].

At the end of trial, samples from the three different microbiomes (water, WATER; skin, SK; anterior intestine, AI) were obtained. WATER samples were taken from water flow entry to the IATS facilities, before entering in contact with the tank (inlet water; IW) or from the outlet flow exit of tanks with fish from each experimental diet. Three aliquots of 1 L of water were taken from IW and each tank (15 L in total; 3 per experimental group). Water was taken just before the usual first feeding of the day using sterile glass flasks. Such water samples were immediately filtered using mixed cellulose ester (MCE) membranes with a pore size of 0.22 μm (Millipore, Germany) under vacuum conditions. In parallel, nine overnight-fasted fish per diet – three per tank – were randomly sampled for AI and SK microbiota studies. SK mucus samples were obtained by gently scrubbing the surface of the fish skin with a sterile microscope slide in favour of the scales, and then transferred to sterile 1.5 mL tubes and stored at − 80 °C until DNA extraction. A portion of the AI (~ 2 cm) was opened and washed with sterile Hank’s balanced salt solution to discard non-autochthonous bacteria, collecting only adherent bacteria by scraping off intestinal mucus with the blunt edge of a sterile scalpel. To avoid contamination, sampling operators were not worn orange clothing (i.e., the colour of the RFs) in the area and microbiota procedures were performed under sterile conditions.

### Bacterial DNA extraction

Bacterial DNA from 72 samples (36 AI and 36 SK samples; 9 samples per group) was extracted with High Pure PCR Template Extraction Kit (Roche, Basel, Switzerland), following the manufacturer’s instructions, including a prior lysis step with lysozyme at a concentration of 250 µg/mL for 15 min at 37 °C [44]. The purified DNA samples were stored at −20 °C until sequencing. Water-associated microorganisms DNA (15 WATER samples; 3 per group) was extracted using the DNeasy PowerSoil Pro Purification Kit (Qiagen, Hilden, Germany). Filters were cut into small fragments and placed into bead-beating lysis tubes provided in the kit. Mechanical lysis was performed using a FastPrep 24 homogenizer (MP Biomedicals, Irvine, CA, USA) at 6 m/s for 30 s. The subsequent extraction steps followed the manufacturer’s instructions. The concentration and quality of the DNA from all samples was assessed using a NanoDrop 2000c (Thermo Fisher, Waltham, MA, USA), and agarose gel electrophoresis (1% w/v in Tris-EDTA buffer).

From the extracted DNA, the hypervariable V1-V3 region of the 16 S rRNA gene (506 bp) was amplified using the corresponding primers (27 F: 5′-AGA GTT TGA TCM TGG CTC AG-3′; 533R: 5′-TTA CCG CGG CKG CTG GCA CG-3′. PCR was prepared in a final volume of 25 µL, using 300 ng of template DNA and a primer concentration of 0.4 µM. The reaction was performed using the LongAmp Hot Start Taq 2× Master Mix (New England Biolabs) setting up the same conditions than in Domingo-Bretón et al. [43]. Along with the samples, a positive control (ZymoBIOMICS™ Microbial Community Standard II, Log Distribution; Zymo Research, USA) and a negative control were included and processed in parallel throughout the entire workflow, including PCR, library preparation, and sequencing. The positive control showed good taxonomic representation, with a strong Pearson correlation coefficient (*r* = 0.953, *p* < 0.001) between expected and observed relative abundances. Amplicons were purified using AMPure XP magnetic beads (Backman Coulter, Brea, CA, USA) with a beads/sample ratio of 0.6, and eluted in a final volume of 30 µL of water. All samples were then quantified by fluorescence using the PicoGreen™ kit (Thermo Fisher, Waltham, MA, USA), and the presence of the specific ~ 500 bp band was verified by agarose gel electrophoresis (1.5% w/v in Tris-EDTA buffer).

### ONT sequencing

Library preparation for sequencing was performed using the Native Barcoding Kit 96 V14 (Oxford Nanopore Technologies, Oxford, UK). All PCR products were diluted equimolarly to a final concentration of 200 femtomoles in a total volume of 12.5 µL. Next, end repair of the sequences was carried out using the NEBNext^®^ Ultra™ II End Repair/dA-Tailing Module (New England Biolabs, Ipswich, MA, USA), following the manufacturer’s instructions. Then, in a first ligation reaction, unique barcoding sequences were added. Once this step was completed, the sequences from each sample were molecularly identified, allowing all samples to be pooled into a single tube. Subsequently, a purification step using magnetic beads was performed, followed by a second ligation reaction to attach the sequencing adapters. Another purification step was carried out after the second ligation, and the final DNA concentration in the library was quantified by fluorescence using PicoGreen.

Library sequencing was performed using the MinION™ sequencing device from the Oxford Nanopore platform. A total of 35 femtomoles of library were loaded onto an R10.4.1/FLO-MIN114 flow cell. The acquisition of raw sequencing data and the demultiplexing process (identification of the barcodes for each recorded sequence) were carried out using the MinKNOW software v23.07.8. The basecalling process (assignment of the corresponding base to each position) was performed using the Dorado v0.7 (https://github.com/nanoporetech/dorado; last accessed: 25 August 2024) algorithm with the most accurate model developed to date (SUP model).

### Bioinformatic analysis

Basecalled reads were then demultiplexed and trimmed from barcodes and adapters using Dorado v0.7. Resulting BAM files were converted into FASTQ format using samtools v1.10 [45], and uploaded to the Sequence Read Archive (SRA) under the BioProject accession number PRJNA1282057 (BioSample accession numbers: SAMN49609176-262). Raw reads were filtered using Chopper v.0.8.0 [46], setting a length threshold of 300–600 bp and quality-filtered by q = 15. Remaining reads were then taxonomically assigned with minimap2 v2.28-r1209 [47] against SILVA v138.1 as a reference database [48]. Picrust2, embedded in SAMBA tool [49], was used to normalize the amplicon data by 16 S rRNA gene copy number and to infer metagenomic content [50]. A 97% sequence identity cut-off was applied, and predicted metagenomic functions were annotated using the Kyoto Encyclopedia of Genes and Genomes (KEGG, October 2018 release) and MetaCyc (v28.5, August 2019 release) databases. Differential pathway abundance was analysed using DESeq2 with default parameters [51]. Because functional profiles were inferred from 16 S rRNA gene data using PICRUSt2, the resulting predictions represent potential metabolic capabilities rather than measured functional activity, and should therefore be interpreted with caution. In the AI analyses, we filtered PICRUSt2 results to functions present at a reasonable prevalence (≥ 75% of AI samples) to avoid noisy predictions. Then, we calculated the average expected number of copies of that gene, normalized by 16 S abundance, in all the AI samples. Pathways in the third quartile or more of the average values distribution were selected and considered our stable and dose-insensitive final functions.

### Statistical analysis

Beta diversity across microbiomes was tested with permutational multivariate analysis of variance (PERMANOVA), using the non-parametric method *adonis* from the R package *vegan* with 10,000 random permutations (https://cran.r-project.org/package=vegan). Microbial community structure among gut, skin and water microbiomes was analysed using non-metric multidimensional scaling (NMDS) based on Bray–Curtis (B-C) dissimilarity matrices calculated from relative abundance data. The NMDS ordinations (stress < 0.2) were computed using the *metaMDS* function in the *vegan* R package. Group dispersion was visualized using 95% confidence ellipses based on Mahalanobis distances. Centroids for each group were calculated, and Euclidean distances between centroids were retrieved between the three microbiomes. Principal Component Analysis (PCA) was performed in R using the *prcomp* function from the *stats* package, with centred and scaled data. Rarefaction curves, species richness estimates, and alpha diversity indices were obtained using the *phyloseq* package for R [52]. To determine statistical differences in species richness, alpha diversity indices, and relative abundance at the phylum level, the Kruskal-Wallis test with the Dunn’s post-test (*p* < 0.05) was employed. For detailed microbiota differences between groups, a discriminant analysis using partial least squares (PLS-DA) was performed with EZinfo v3.0 software (Umetrics Umeå, Sweden). The Hotelling’s T2 statistic was calculated, and points that exceeded the 99% confidence limit were considered outliers and excluded from the model. The quality of the PLS-DA model was assessed using the R2Y (cum) and Q2 (cum) parameters, which indicate the ability of the model to fit and predict group differences, respectively. The contribution of different bacterial genera to group separation was determined using the variable importance in projection (VIP) value, fixing a minimum VIP threshold that cluster > 90% of the experimental groups samples to identify discriminative variables in the PLS-DA model [53]. The most abundant discriminant bacteria were defined at a 1% relative abundance. The inferred metagenomics pathways were considered differentially represented using an FDR-corrected significance threshold of 0.05. Spearman correlation coefficients (CC) were calculated between the normalized abundances of discriminant OTUs in the samples and the normalized multi-tissue gene expression values reported in Matias et al. [23], averaged per tank. The corresponding p-values were obtained using the cor.test function from the corrplot R package, with a two-sided alternative hypothesis. Significant gene–OTU correlations were defined as those with *p* < 0.01 and |CC| > 0.6, and were visualized using Cytoscape v3.8.2.

## Results

### RF intake modulates Multi-Microbiome similarities within the European sea bass holobiont

**Fig. 1:**
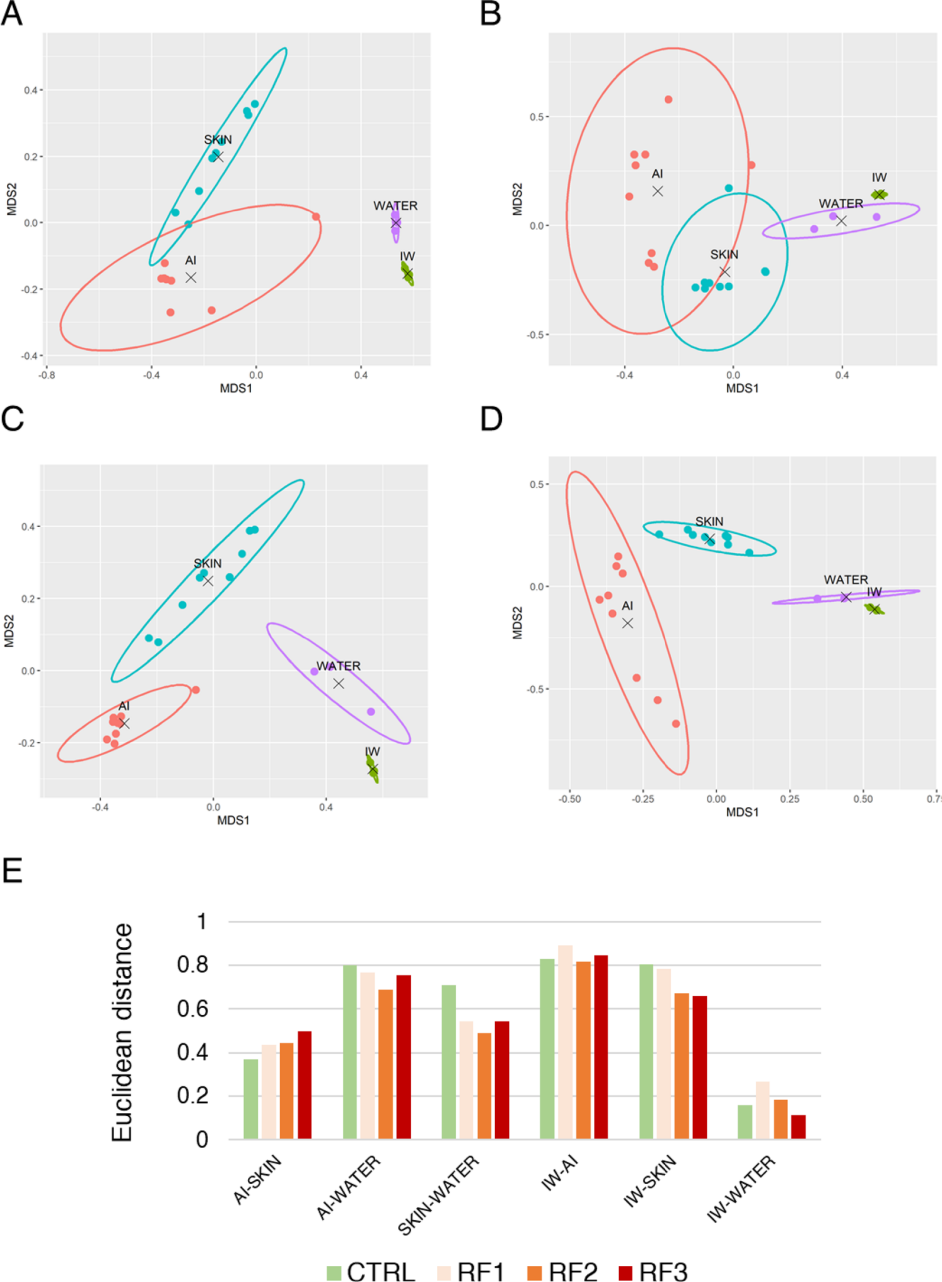
Non-metric multidimensional scaling (NMDS) ordination plot based on Bray–Curtis dissimilarity of relative microbial abundances in **A** CTRL, **B** RF1, **C** RF2 and **D** RF3, illustrating the community structure among gut, skin, and water microbiomes. Stress values were < 0.2, indicating a reliable two-dimensional representation. Ellipses represent 95% confidence intervals calculated using Mahalanobis distances. Centroids denote the average community position for each microbiome type. **E** Bar plots representing the Euclidean distances between centroids, reflecting compositional dissimilarity across compartments

ONT sequencing and read filtering steps produced a total of 8,238,066 amplicon reads, averaging 94,690 reads per sample that were taxonomically assigned at a mean rate of 78.3% (Supplementary Table 1), approximating asymptotic saturation after rarefaction analysis (Supplementary Fig. 1). Taxonomy assignment identified 2,818 taxa across the AI, SK and water microbiomes, that showed significant differences in β-diversity (*p* < 0.001; F = 1.913, R^2^ = 0.089). Almost 90% of these taxa were classified up to the level of genus, with increasing percentages to the levels of family (95.1%), order (97.5%), class (98.8%), and phylum (99.9%). After this assignment, microbiome structure analysis using NMDS showed a change in the group space disposition with the increase of RF load (Fig. 1A-D). Data from AI and SK datasets displayed the shortest Euclidean distances (ED) that ranged between 0.4 and 0.5 regardless of the diet (Fig. 1E). Interestingly, the distance between SK and WATER microbiomes was shortened with the RF intake, peaking at 0.71 amongst the CTRL data, and shifting down to 0.544, 0.488 and 0.545 in RF1, RF2 and RF3, respectively. In comparison, the AI and WATER microbiomes showed the highest distance values (0.73–0.79) across the RF groups. The inclusion of IW group showed a higher degree of similarity with WATER (0.113–0.266 Euclidean distance) than with AI (> 0.81) and SK (> 0.65).

### Measures of α-diversity and phylum composition revealed a convergence between RF-free and RF-high-dose diets

**Fig. 2:**
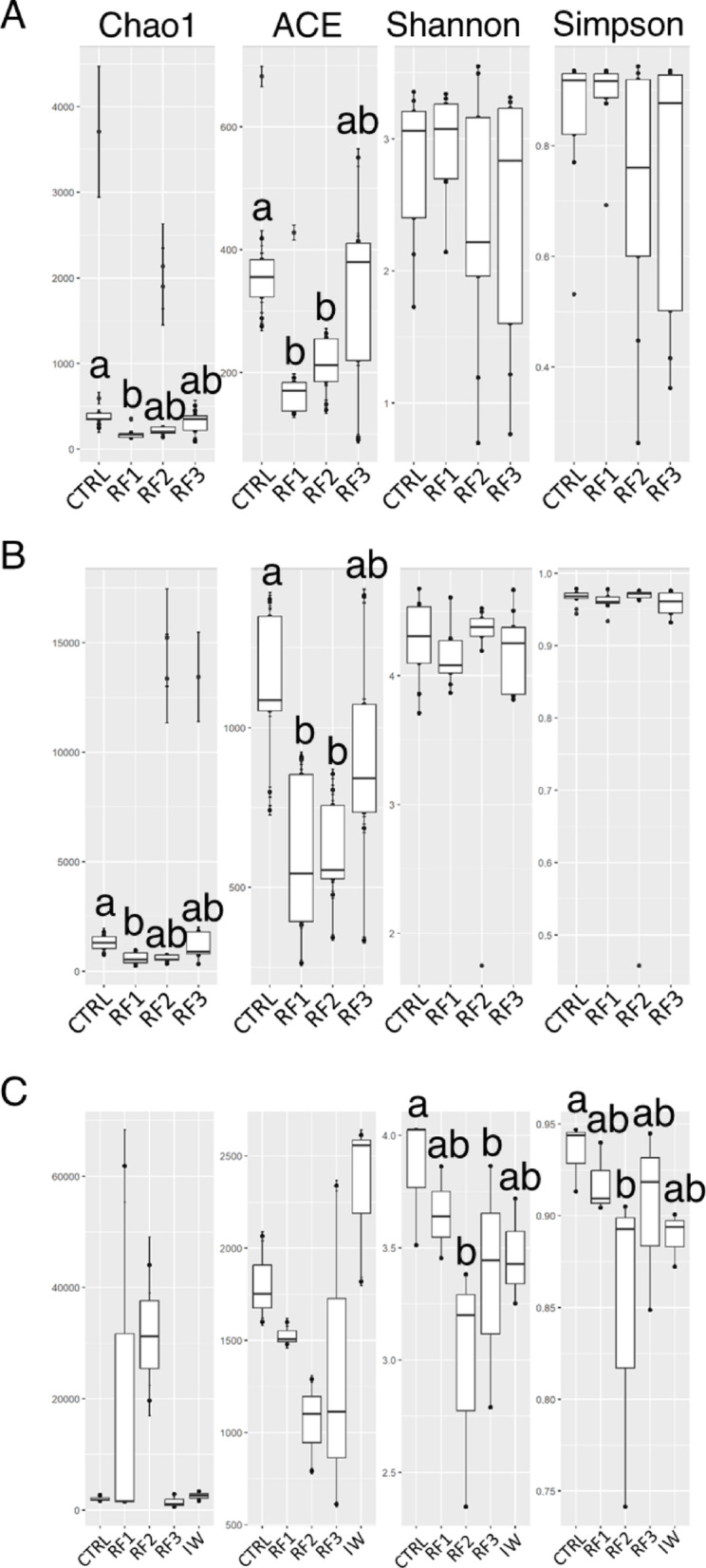
Boxplots representing richness estimators (Chao1 and ACE) and diversity indexes (Shannon and Simpson) of **A** AI, **B** SK and **C** WATER microbiomes across RF-dietary groups. Different letters indicate significant differences among groups (Kruskal-Wallis test with Dunn’s post-test, *p* < 0.05)

When comparing the bacterial α-diversity across the four dietary groups, a clear dichotomy between richness and evenness emerged among the different microbiota compartments (Fig. 2). The richness indices (Chao1 and ACE) in AI and SK exhibited a similar trend: highest values were observed in the CTRL group, followed by a decrease in RF1 and RF2, and a subsequent increase in the RF3 group (Fig. 2A, B). In contrast, the WATER microbiome showed no significant richness differences among groups. However, evenness indices (Shannon and Simpson) were only significantly reduced in the RF2 and RF3 groups of WATER compared to the CTRL group (Fig. 2C), indicating compositional imbalance under higher RF exposure.

**Fig. 3:**
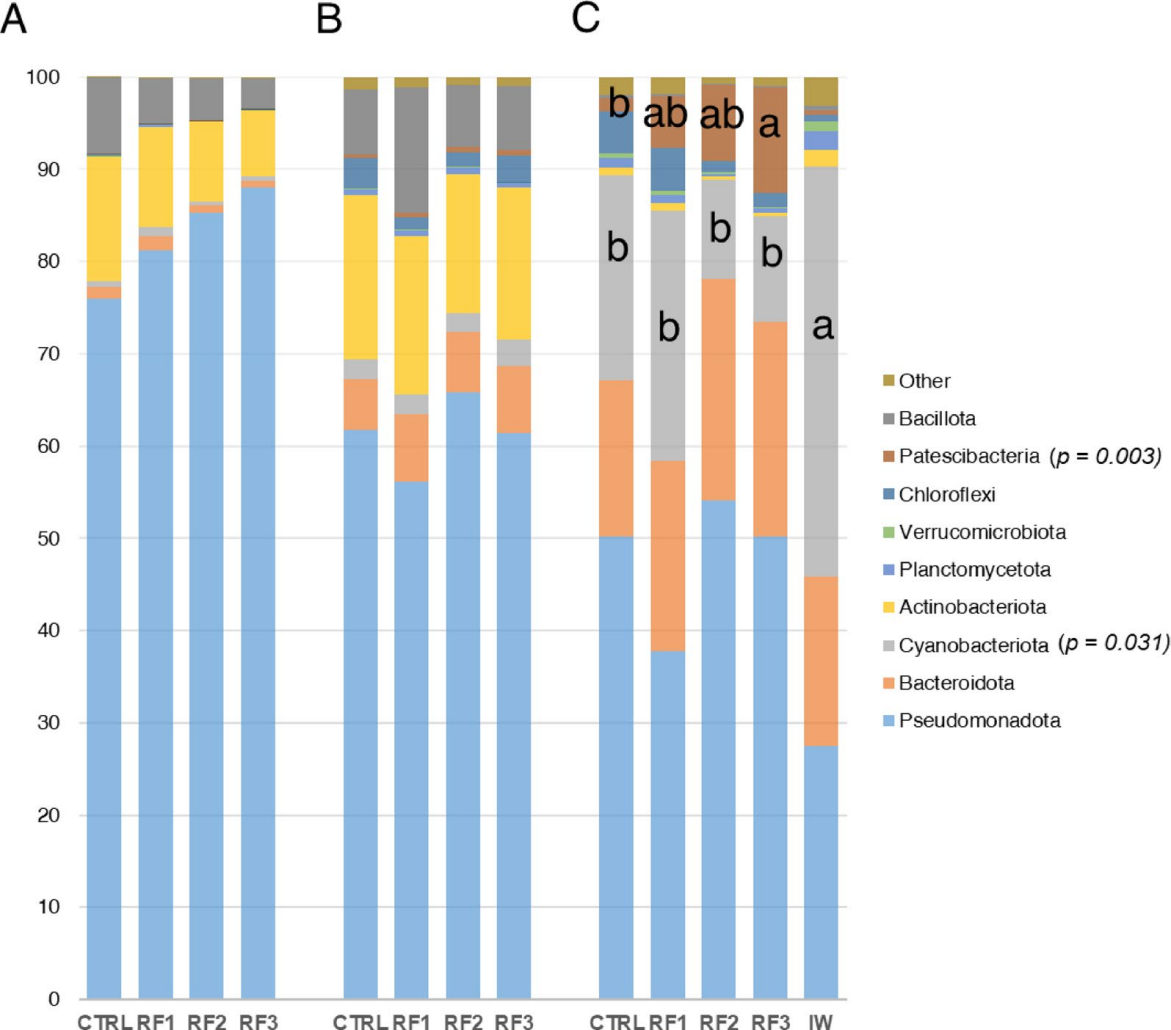
Stacked bar chart representing the relative abundance of microbial taxa in **A** AI, **B** SK and **C** WATER microbiomes between RF-dietary groups. Different letters indicate significant differences among groups (Kruskal-Wallis test with Dunn’s post-test, *p* < 0.05)

At the phylum level, no statistically significant differences were detected in the bacterial composition of AI and SK) microbiomes among dietary groups, based on Kruskal-Wallis and Dunn’s post-hoc tests (Fig. 3A, B). However, a progressive increase in the relative abundance of Pseudomonadota (from 76% to 88%) was observed with the increasing dietary RF load, accompanied by a corresponding decrease in Actinobacteriota and Bacillota (Fig. 3A). Specifically, Pseudomonadota was the most abundant phylum, constituting > 75% of the total resident bacteria in the AI, and > 55% in the SK. Actinobacteriota was the second most abundant, slightly more prevalent in the SK (> 15% in all groups) than in the AI (> 10%). Bacillota also represented a substantial fraction, ranging from 5 to 10% in the AI and 8–15% in the SK. Notably, Bacteroidota showed compartment-specific differences: it represented less than 2% of the AI microbiota, but approximately 10% of the SK microbiota across all groups. Overall, WATER microbiome composition exhibited a distinct phylum-level composition compared to AI and SK (Fig. 3C). In this compartment, Pseudomonadota, although maintained as the top abundant phylum, was below 55% in all dietary groups, whereas Actinobacteriota and Bacillota were diffused within the plot (< 2%). Among the most abundant phyla in water, Bacteroidota ranged from 15 to 22%, and Planctomycetota from 3 to 5% across the experimental groups. Notably, Patescibacteria displayed a marked increase in response to RF exposure, rising from 2% in the CTRL group to 14% in the RF3 group. This phylum was nearly absent (< 1%) in the IW samples (water without fish), indicating its association with fish-derived microbiome shifts. The presence of fish also significantly influenced Cyanobacteriota, whose abundance declined from 40% in the IW to 10–20% across the CTRL to RF3 groups.

### Discriminant analyses showed a concomitant dose-dependent and dose-threshold microbial responses to RFs ingestion

**Fig. 4:**
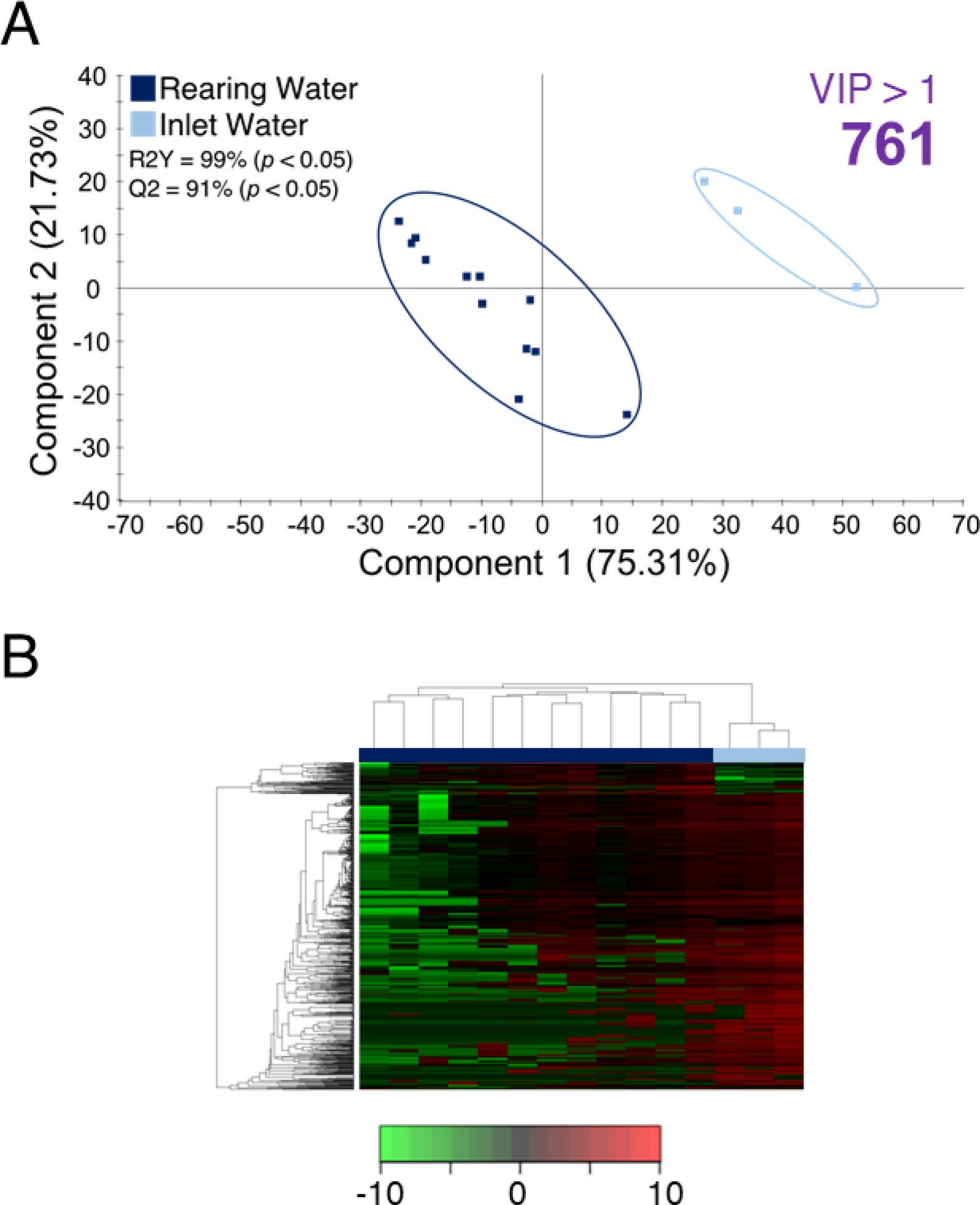
**A** Two-dimensional representation of the distribution of samples between the two first components of partial least squares discriminant analysis (PLS-DA) driving the separation of the Rearing (CTRL_RF1_RF2_RF3) and Inlet (IW) microbiota, in order to disclose the effects of fish presence in the tanks, regardless the RF dose. Right-top purple bold numbers are the number of taxa surpassing the necessary VIP value to accomplish the group separation. *P*-values are the result after 500 permutation validation tests. **B** Heatmaps represent the abundance distribution (Z-score) of the OTUs identified to be driving the separation by diet

**Fig. 5:**
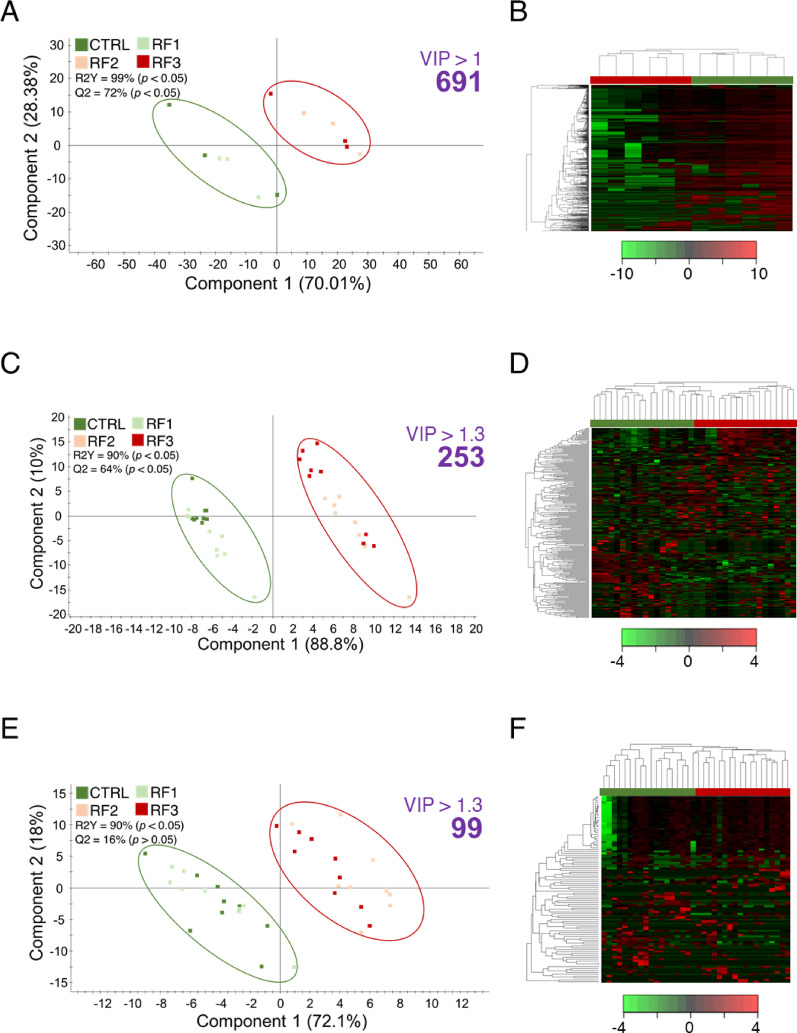
Two-dimensional representation of the distribution of dose-dependent RFs samples between the two first components of partial least squares discriminant analysis (PLS-DA) driving the separation of **A** WATER, **B** SK and **C** AI microbiomes across RF-dietary groups. Right-top purple bold numbers are the number of taxa surpassing the necessary VIP value to accomplish the group separation. *P*-values are the result after 500 permutation validation tests. Heatmaps represent the abundance distribution (Z-score) of the OTUs identified to be driving the separation by diet in **D** AI, **E** SK and **F** WATER microbiomes, respectively

**Fig. 6:**
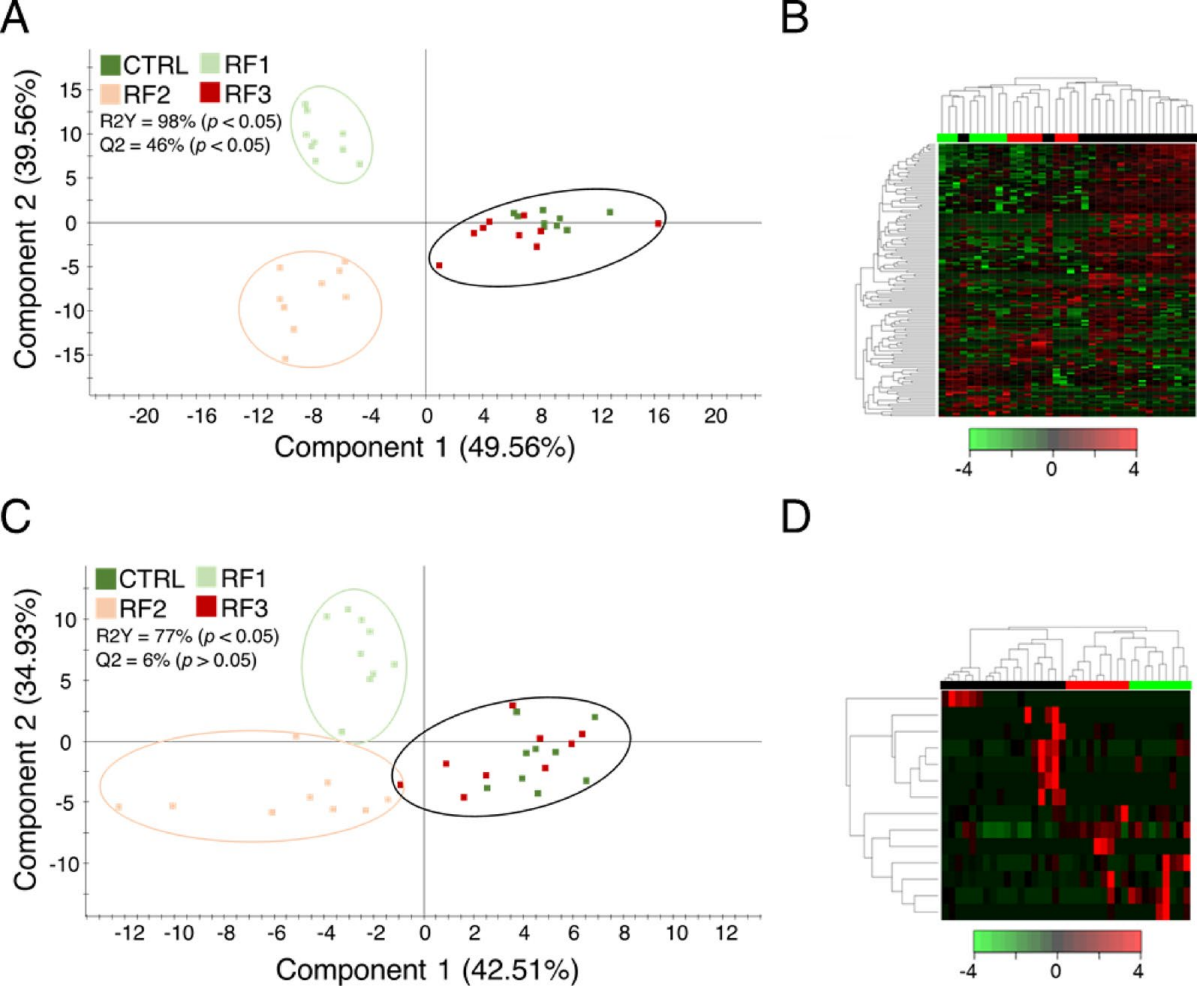
Two-dimensional representation of the distribution of samples between the two first components of partial least squares discriminant analysis (PLS-DA) driving the separation of the gradual (i.e., those taxa increasing in RF1 and RF2 and returning to CTRL values in RF3) of **A** SK and **B** AI microbiomes. Right-top purple bold numbers are the number of taxa surpassing the necessary VIP value to accomplish the group separation. *P*-values are the result after 500 permutation validation tests. Heatmaps represent the abundance distribution (Z-score) of the OTUs identified to be driving the separation by diet in **C** AI and **D** SK microbiomes, respectively

To corroborate and explore phylum-level differences in more detail, various PLS-DA models were constructed and statistically validated (Figs. 4, 5 and 6). A first PLS-DA separating Rearing Water (CTRL_RF1_RF2_RF3) and IW confirmed the strong effect of the presence of fish in the tank on aquatic microbiota (R2Y = 99%; Q2 = 91%; pR2Y < 0.05; pQ2 > 0.05; Fig. 4A), clustering perfectly the two experimental groups (Fig. 4B). To compare the effect of plastic low and high doses in each compartment, three PLS-DA (one per compartment) were constructed (Fig. 5). The WATER PLS-DA model revealed a very significant group separation (R2Y = 99%; Q2 = 72%; pR2Y < 0.05; pQ2 < 0.05), indicating a strong discriminative power (Fig. 5A). This adjusted model identified 691 discriminant taxa (46% of taxa inputted to the analysis; VIP > 1), driving a correct hierarchical clustering that grouped all samples according to their respective treatment (Fig. 5D). The same group comparison and disposition (R2Y = 90%; Q2 = 64%; pR2Y < 0.05; pQ2 < 0.05) was observed for the SK PLS-DA (Fig. 5B), after a VIP cut-off of 1.3 that revealed 253 taxa (18% of the taxa inputted to the analysis) of discriminant value, as confirmed by heatmap clustering (Fig. 5E). With the same VIP threshold, we identified up to 99 taxa (17% of the taxa inputted to the analysis) of discriminant value that clustered correctly all samples in the corresponding group (Figs. 5C, F), though the resulting PLSDA model was able to statistically validate the observed variance but not the predicted one (R2Y = 90%; Q2 = 16%; pR2Y < 0.05; pQ2 > 0.05). Altogether, these results indicate a gradual response from AI to WATER, according to which the AI-adherent bacterial communities were the most resilient, while WATER communities experienced the strongest dose-dependent modulation by RF exposure.

Next, we wanted to deepen into the mechanisms underlying host-associated microbiomes responses. To do this, an exploratory PCA with all assigned taxa informed of a possible grouping of CTRL and RF3 samples into a single cluster (CTRL_RF3) in AI and SK microbiomes (Supplementary Fig. 3 A, B), being maintained the dose-dependent effect in WATER microbiome (Supplementary Fig. 3 C). This trend was further confirmed by PLS-DA analyses, which separated CTRL_RF3, RF1, and RF2 groups, but with different statistical meanings (Fig. 6). A statistically significant separation was achieved in SK (R2Y = 98%; Q2 = 46%; pR2Y < 0.05; pQ2 < 0.05; Fig. 6A), whereas low variance metrics were accomplished in AI (R2Y = 77%; Q2 = 7%; pR2Y < 0.05; pQ2 > 0.05), reinforcing the enclosed nature of gut microbiome (Fig. 6B). From this microbiomes disposition, 141 SK (VIP > 1.3) and 13 AI (VIP > 1.7) discriminant taxa were prioritized after clustering (Fig. 6C, D). Discriminant taxonomies driving the separation of groups in Figs. 5A and 6A and B, which will be further discussed, can be accessed in Supplementary Table 2.

### Abundance filters disclosed important microbial fluctuations in the holobiont of the European sea bass

**Fig. 7:**
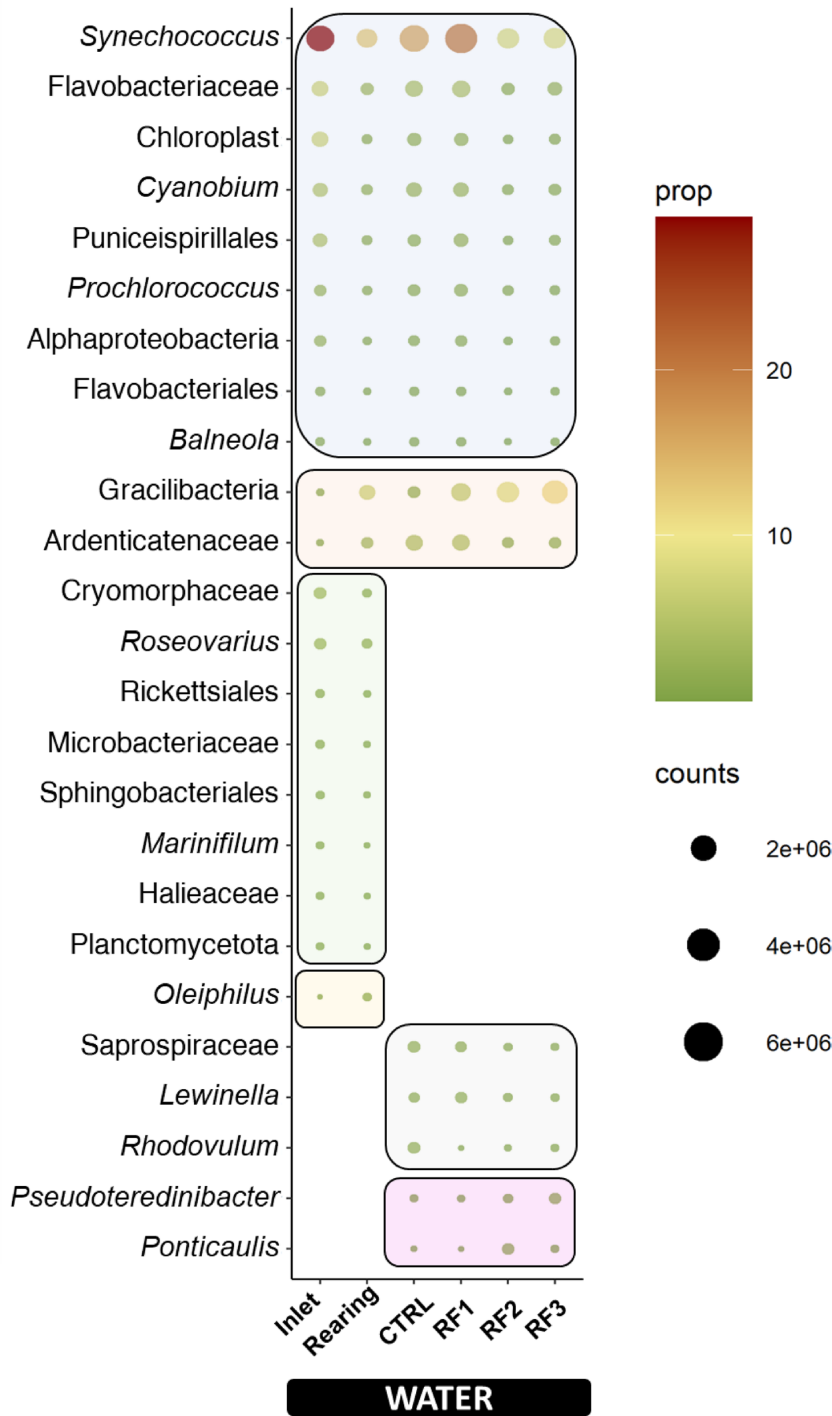
Dot plot representing discriminant taxa (VIP > 1) with relative abundances > 1% in at least one group of Inlet (IW) and Rearing (CTRL_RF1_RF2_RF3) water microbiota (results from PLS-DA separation in Fig. 4A) and dose-dependent (CTRL, RF1, RF2 and RF3) microbiota (results from PLS-DA separation in Fig. 5A)

**Fig. 8:**
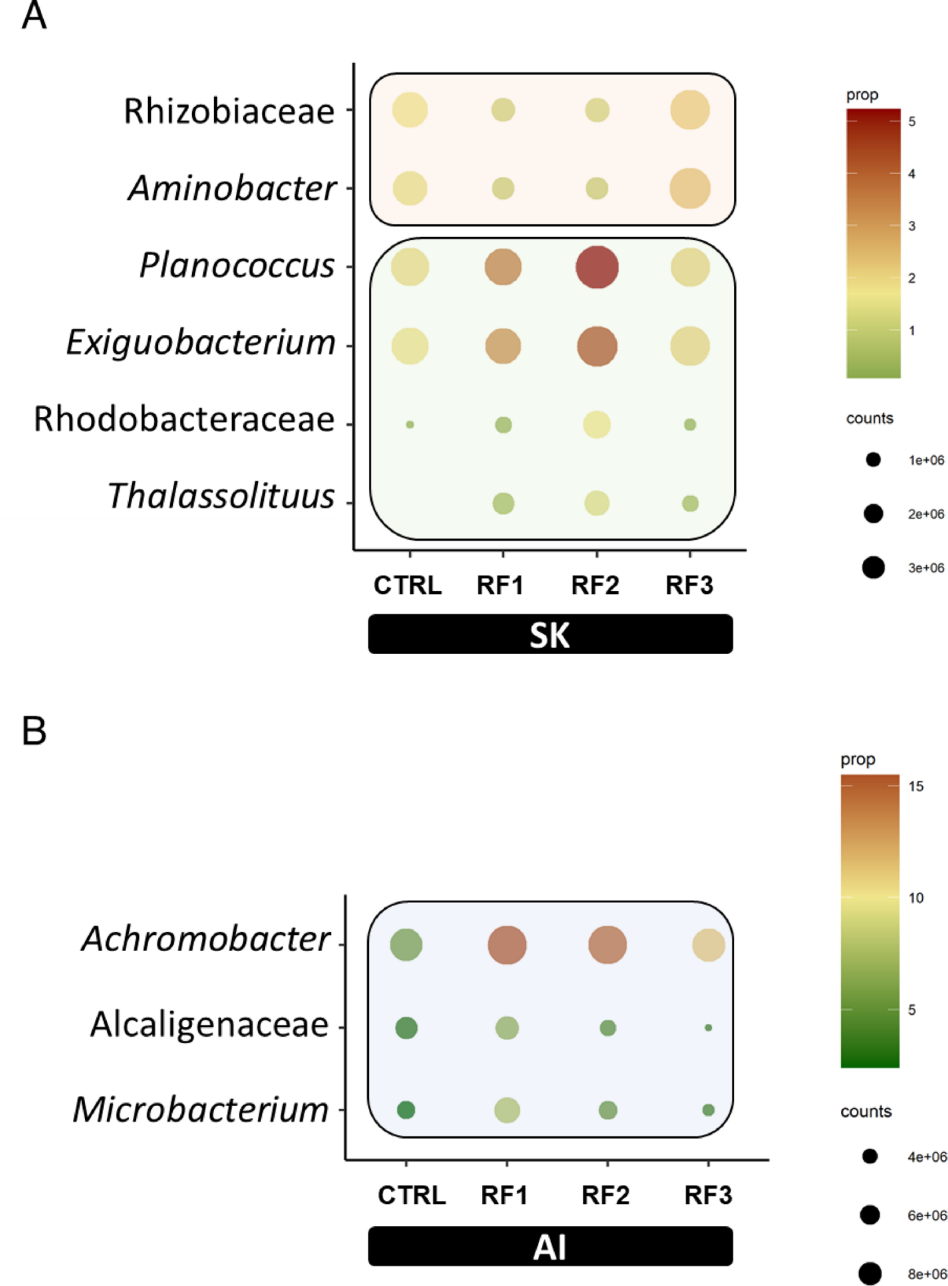
Dot plot representing discriminant taxa (VIP > 1) with more than 1% of relative abundance in at least one experimental group of **A** AI and **B** SK microbiomes. Colour scale represents the mean relative abundance, in percentage, of each taxon within each group. Size of the dots represents normalized counts in each group

The most abundant bacteria (> 1%) of those that exclusively drove the separation by diet in the different microbiomes are presented in Figs. 7 and 8. In WATER PLS-DAs, a total of 25 discriminant taxa were relatively abundant in our experimental groups (Fig. 7). Of them, 9 taxa (*Synechococcus*, Chloroplast, *Cyanobium*, Puniceispirillales, *Prochlorococcus*, Alphaproteobacteria and Flavobacteriales, *Flavobacteraceae* and *Balneola*) were concurrently decreased due to fish presence and increasing RFs concentration in the feed. Interestingly, *Synechococcus* genus, the most predominant taxa in IW (29%), decreased progressively with both the presence of fish and RF exposure (average CTRL and RF1, 17.8%; average RF2 and RF3, 8.1%). Otherwise, two taxa (*Ardenticatenaceae* and Gracilibacteria) followed the contrary pattern, increasing with fish presence and RF dose (IW, 0.36%; CTRL_RF1. 3.4%; RF2_RF3, 9.9%). Eight more taxa (*Cryomorphaceae*, *Roseovarius* Rickettsiales, *Microbacteriaceae*, Sphingobacteriales, *Marinifilum*, *Haelieaceae* and Planctomycetota) decreased exclusively with fish presence, while only *Oleiphilus* genus increased in the Rearing water. With a higher RF dose, a decrease in the *Saprospiraceae*, *Lewinella* and *Rhodovulum* taxa was in parallel to an increase in the waterborne *Pseudoteredinibacter* and *Ponticaulis* genera.

In SK, we identified 6 abundant bacteria driving the separation between groups (Fig. 8A). Two *Rhizobiaceae*-related taxa (*Rhizobiaceae* and *Aminobacter*) were higher in CTRL and RF3 groups (3.5%), and decreased in RF1 and RF2 (~ 1%). Furthermore, up to 4 taxa (*Rhodobacteraceae*, *Planococcus*, *Exiguobacterium*, *Thalassolituus*), although relatively abundant in CTRL group (4.5%; 0.2–2.2%), were clearly found to be predominant in the RF2 group (12.7%; 1.4–5.2%). In AI, abundant threshold highlighted three bacterial taxa with high (> 3%) relative abundances in all of the groups (Fig. 8B). These were *Alcaligenaceae* family (CTRL and RF3, 3–3.7.7%; RF1. 6.5%; RF2. 4.5%), and *Achromobacter* (CTRL, ~ 5.5%; RFs. 11.3–15.5%) and *Microbacterium* (CTRL and RF3, 2.3–3.7%; RF1 and RF2, 5–7.7.7%) genera. The sum of these relative abundances clearly indicated an a higher in CTRL (10%) than in RF-based groups (18–29%). Notably, there was no overlap in the abundant discriminant taxa identified across the three microbiomes and the distinct comparisons, indicating distinct compartment-specific microbial responses.

### Inferred metagenomes highlighted hydrocarbon, carbohydrates and vitamin metabolism

**Fig. 9:**
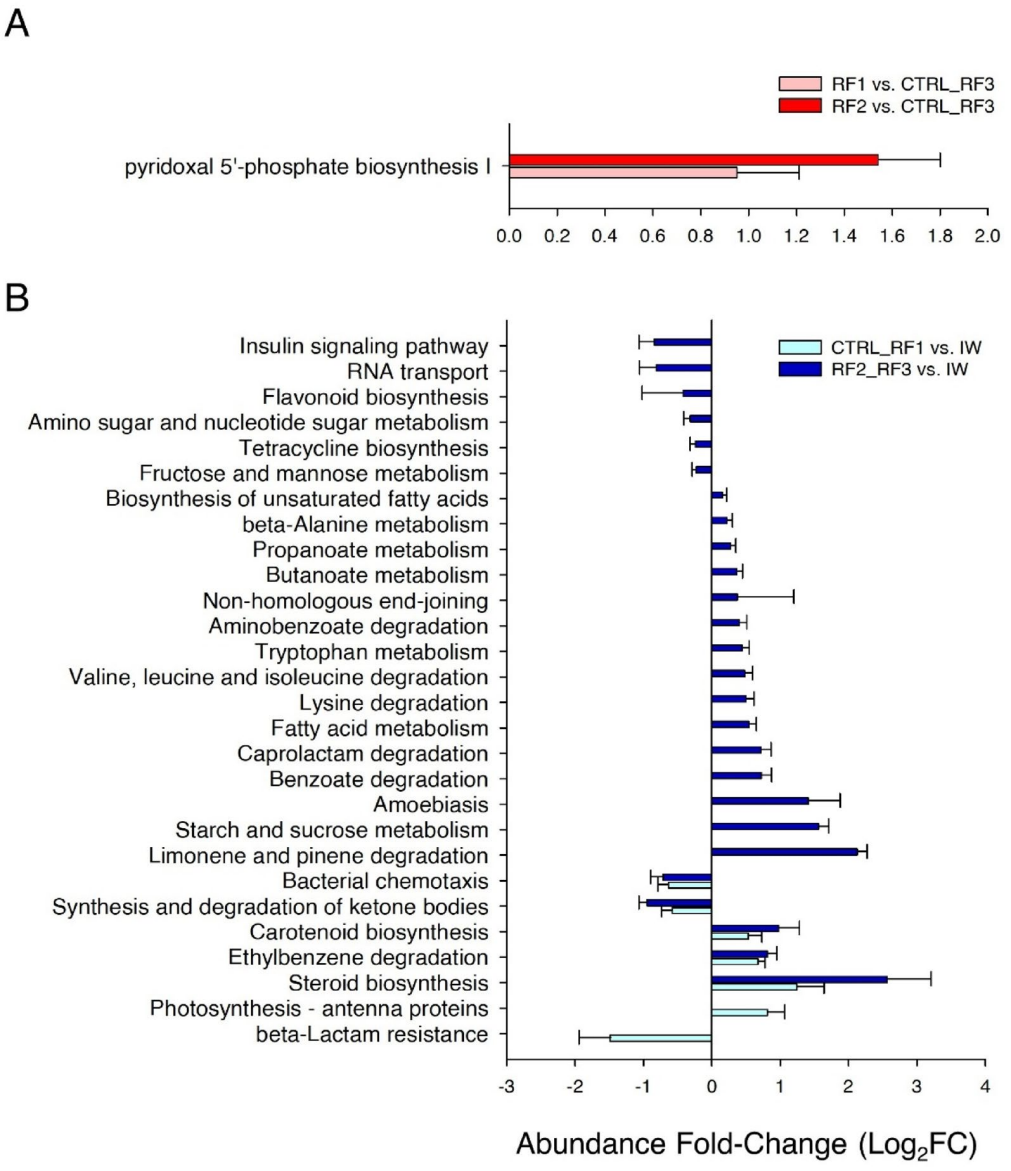
Bar plots depicting the changes in metabolic capacities in the comparison between RFs groups discriminated in the PLS-DA of **A** SK, **B** WATER. Bars show the log_2_fold change in the metabolic pathway

To evaluate the biological relevance of diet-induced differences in the microbiota across groups, inferred pathway analysis was performed using the predicted metagenomes of taxa identified as diet-discriminant in each microbiome (Figs. 5A and 6A and B). In AI, due to the lower number of discriminant taxa, no pathway was discovered as significant. Consequently, 41 inferred pathways could be described as stable and dose-insensitive functions in our AI samples (Supplementary Table 3). These functions were mainly related with Carbohydrate metabolism (8), Metabolism of cofactors and vitamins (8), and Amino acid metabolism (6). Otherwise, in SK, pyridoxal 5’-phosphate biosynthesis I was over-represented in RF1 and RF2 groups (Fig. 9A). In water, 28 KEGG and METACYC categories were differentially represented, outperforming the number of over- and under-represented pathways of the other microbiomes (Fig. 9B). Among the under-represented pathways consistently changing in the presence of RFs vs. IW, there were pathways related to Bacterial chemotaxis and Synthesis and degradation of ketone bodies. Meanwhile, over-represented pathways under the same comparison were related with Carotenoid biosynthesis, Ethylbenzene degradation and Steroid biosynthesis. Interestingly, in RF2_RF3 group, starch and sucrose metabolism and limonene and pinene degradation were both up-regulated, while insulin signalling pathway, RNA transport and flavonoid biosynthesis were the most under-regulated categories. Of note, these results were obtained using prediction software and they only reflect the metabolic potential of these populations.

### Correlation analyses with transcriptomic data reinforces the association of gut bacteria and inflammatory markers

**Fig. 10:**
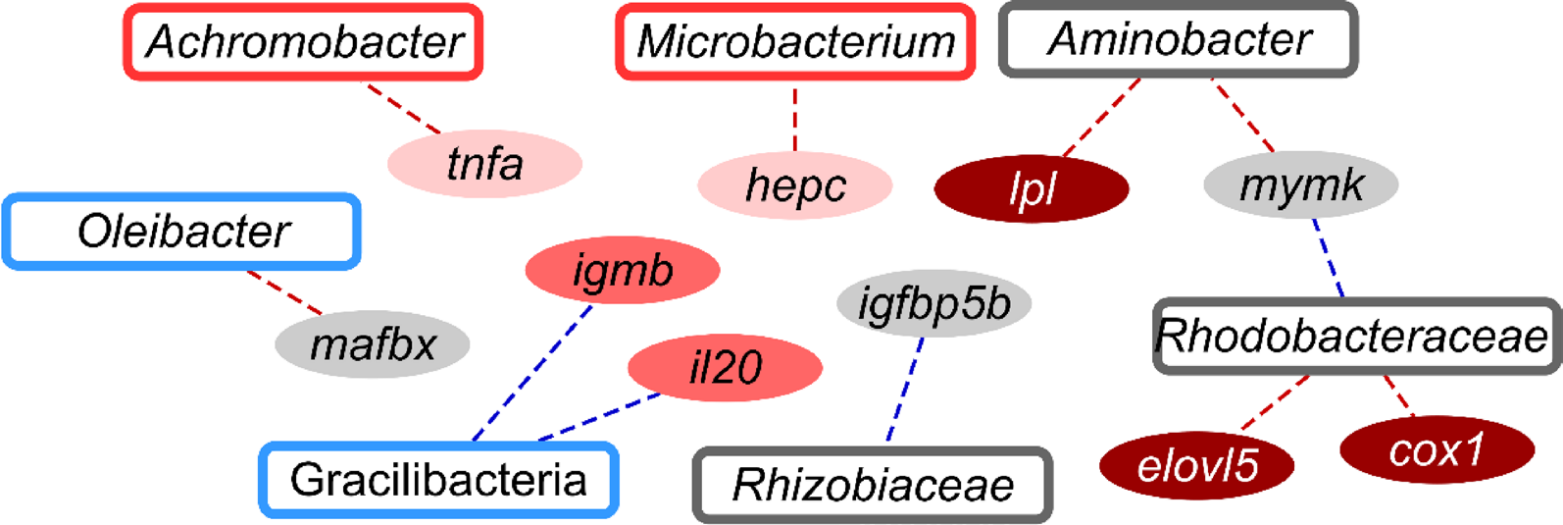
Correlation network showing significant positive (red dashed lines) and negative (blue dashed lines) correlations (Spearman, *p* < 0.05, |CC| > 0.6) between discriminant taxa in AI (red-border rectangles), SK (grey-border rectangles), and WATER (blue-border rectangles) microbiomes, and intestinal (light pink), hepatic (brown), muscular (grey), and head-kidney (dark pink) genes

In total, 1,258 correlations were performed between discriminant taxa from the three microbiomes and gene expression data for all genes studied in Matias et al., 2025 [23], establishing 11 significant associations (*p* < 0.05, |CC| > 0.6) between 7 discriminant OTUs and 10 DE genes (Supplementary Table 4; Fig. 10). Of note, *Achromobacter* and *Microbacterium* abundances in gut were positively correlated with the tumour necrosis factor alpha (*tnfα*) and hepcidin precursor (*hepc*) gene expression data, respectively. Skin bacteria abundances of the *Aminobacter* genera were correlated positively with myomaker (*mymk*) gene, whereas *Rhizobiaceae*, and *Rhodobacteraceae* taxa showed a negative correlation with *mymk* and insulin growth factor binding protein 5 (*igfbp5*). To complete the correlation list, positive links were found between *Aminobacter* and *Rhodobacteraceae* skin taxa and the hepatic lipoprotein lipase (*lpl*), cytochrome c oxidase subunit I (*coxi*) and elongation of very long chain fatty acids 5 (*elovl5*). The increase of Gracilibacteria in the rearing water microbiome was associated negatively to interleukin-20 (*il20*) and immunoglobulin m membrane bound (*igmb*) gene expression in head-kidney, whereas WATER *Oleibacter* was positively associated to muscle atrophy F-box (*mafbx/atrogin 1*).

## Discussion

Understanding how host-associated and environmental microbial communities respond to anthropogenic pressures is increasingly recognized as essential for maintaining ecosystem balance in aquatic organisms [54]. Accordingly, the present study applied a holobiont framework to examine microbiome responses across intestinal, cutaneous, and surrounding water microbiomes in farmed fish exposed to graded dietary RF loads. Notably, the three bacterial compartments exhibited distinct compositions, highlighting compartment-specific dynamics under RF exposure. This is not surprising as it is well established that gut, skin, and water microbiomes differ substantially in terms of diversity, taxonomic composition, and environmental interactions [55–57]. Indeed, our NMDS analysis revealed that, in the absence of RF exposure, AI and SK and WATER and IW microbiomes exhibited a relatively closer spatial distribution, as reflected by shorter Euclidean distances in the NMDS plot. AI and SK similarity can be likely due to both being host-associated microbiomes, shaped and maintained through host-specific interactions and influenced by intrinsic factors such as immune system activity, physiology, genetics, and lifestyle. Evidence of host dependency is well established for fish gut microbiota [58–62], and also for SK-associated microbiota, which is shaped both by host influences and by external factors such as diet, rearing density, and water oxygen availability [63–65]. Such complexity underpins tissue-specialized host–microbiome interactions, in which the host provides habitat and nutritional foundations, while the microbiota expands metabolic capacities by enabling the use of resources otherwise inaccessible to the host alone [66]. Moreover, in the absence of RF exposure, we found herein that the SK microbiome did not occupy an intermediate position between AI and WATER. However, with increasing dietary RFs, the projection shifted, showing a progressive convergence between SK and WATER microbiomes. Such observation provides evidence that a given environmental stimulus can drive these communities toward greater similarity, at least in terms of their overall structure. The plasticity of the SK microbiome has also been observed in other fish species, as exemplified the shifts shaped by water salinity and geographic location [67, 68]. Likewise, the convergence of SK and WATER bacterial communities have also been observed in gilthead sea bream fed both starch- and non-starch carbohydrate-rich diets [69]. The underlying mechanism driving this convergence remains uncertain, but two hypotheses can be considered. First, RF exposure may alter the water microbial pool or water chemistry, facilitating the direct colonization of the skin by waterborne taxa. Second, dietary RFs may induce host-mediated changes in the skin mucosal environment (e.g., modifications in mucus composition, antimicrobial peptide activity, or epithelial turnover) that reduce the selective filtering normally exerted by the skin and thereby favour the establishment of water-associated bacteria. These possibilities suggest that both environmental seeding and host-driven shifts in habitat suitability may contribute to the observed SK–WATER convergence, although further mechanistic investigation is required.

Previous in vivo and in vitro studies highly support that MP exposure alters microbiota imbalance [70]. From our results, it is also conclusive that dietary RFs led to a decline in microbial richness within the host-associated microbiomes. Such effects likely arise from the dual nature of MPs, which act both as physical stressors and as sources of chemical additives, thereby creating selective pressures that can promote resilient taxa while constraining sensitive or rare microbial populations [33]. Consistent with these selective pressures, a pro-inflammatory state in RF1 and RF2 may also reduce bacterial richness by damaging microbial niches and favouring inflammation-tolerant species [23]. Thus, as found in previous gut microbiota studies in European sea bass [71, 72], the phyla of Pseudomonadota, Actinobacteriota and Bacillota dominated the host-associated niches. These gut abundant phyla were also majorly represented in the SK microbiome, but with a reduced presence of Pseudomonadota that also occurred in a higher extent in the WATER microbiome. Following Pseudomonadota, the Cyanobacteriota phylum was the second most abundant in the WATER microbiome, which is consistent with the intense phototrophic activity occurring in marine environments [73]. Fish presence influence these microbial patterns, likely through changes in water optical properties, sediment resuspension, or the release of particulate waste that increases turbidity, which in turn reduces light penetration and adversely affects Cyanobacteriota activity and survival [74]. Thus, Actinobacteriota, usually present in deep-sea microbiota [75], almost disappeared in the WATER microbiome of our study, facilitating a rise in Bacteroidota that is consistent with the patterns observed in open-water and coastal pelagic microbial communities [76].The rise of Bacteroidota was not associated herein to the exposure of rearing fish to RFs diets. Indeed, the abundance of the Patescibacteria phylum increased progressively in the rearing water of fish exposed to higher dietary RF loads, consistent with previous observations of this taxon in microplastic-polluted waters [77]. These results put a first look into the impact of cellulosic microfibres pollution on aquaculture microbiomes, allowing us to start defining the “rearing water plastisphere”, the group of microorganisms associated with these substances in farming environments, that include the presence of fish [77–79], and Gracilibacteria in particular (see below). This, in turn, led to a WATER microbiome that exhibited the strongest and measurable shifts following RF exposure, whereas the SK and AI microbiomes showed weaker or only marginally significant responses, which would be facilitated by the enclosed nature of gut [36, 80]. Certainly, aquatic environments not only serve as major sinks for plastic pollution [81], but also provide a permissive medium for microbiota proliferation following microplastic and/or MFs pollution [82]. By contrast, living organisms can mount tissue-specific responses against pollutants and environmental hazards to minimize any physiological disturbance, including alterations in the host-associated microbiomes [38, 83].

Keeping in mind the restrictive and distinctive nature of the host-associated microbiomes, we aimed to investigate the response dynamics that protect host fitness and performance. In this query, we discerned a threshold pattern, in which both the AI and SK microbiomes of the CTRL and RF3 groups clustered together. Explaining this apparently contradictory finding is not straightforward. However, several studies have shown that host biological responses are often activated only above a certain MP ingestion threshold [84]. In fact, to evaluate the effects of microplastic (MP) pollution, a Threshold Microplastics Concentration (TMC) framework has been proposed, aiming to define the concentration at which MPs and their associated contaminants begin to pose a concern for animal health [85]. This TMC does not indicate an immediate hazard but instead serves as a signal for risk assessment or intervention, reflecting the activation of regulatory responses in the animal. Microbiome responses are undoubtedly part of these regulatory processes, yet only limited information is currently available for aquaculture fish species [83]. To help fill this gap, our discriminant analyses identified 13 taxa in AI and 141 taxa in SK as highly responsive bacteria communities to RFs pollution, whose physiological significance is detailed below. Of note, these significances must be tempered in AI, where PLS-DA did not arrive to separate the groups correctly. Anyway, after a posterior VIP filtering, clustering was able to group CTRL and RF3, RF1 and RF2, separately. Otherwise, the SK PLS-DA showed a remarkable separation and clustering performance after taxa prioritization.

European sea bass is a typical carnivore with limited ability to digest carbohydrates, as it lacks the enzymes needed to break down these complex molecules [86, 87]. To compensate, it partly relies on intestinal bacteria that can ferment these carbohydrates, helping to improve feed utilization [88, 89]. This symbiotic relationship is supported by polysaccharide-degrading members of the core microbiota, including among others bacterial taxa from Burkholderiales and Micrococcales, which likely contribute to nutrient processing and host-microbe interactions [90, 91]. In our study, these orders are represented by *Alcaligenaceae* family and *Achromobacter* and *Microbacterium* genera. These taxa were highly abundant across all experimental groups, though the highest abundance was achieved in RF1 and RF2 groups instead of RF3, perhaps as part of the adaptive host response that may serve to minimize the negative impact or a host surplus of cellulosic substrates. Indeed, *Alcaligenaceae* taxa carry glycoside hydrolases, glycosidases, and other carbohydrate-active enzymes that break down cellulose, hemicellulose, and xylan [92, 93], while *Microbacterium* species produce cellulases that hydrolyze the β-linked bonds in cellulose [92, 94]. Otherwise, some of these bacteria, particularly *Achromobacter* (the most abundant gut taxon of discriminant value in our study), is recognized as a pro-inflammatory bacteria, capable of triggering the expression level of several cytokines such as *il-6*, *il-8* and *tnfα*, leading to tissue inflammation and damage [95]. This aligns with a local pro-inflammatory condition as the result of the intestinal up-regulation of *il1b* and *tnfα* genes in fish from the same experiment fed with low (RF1) and intermediate (RF2) RF loads [23]. Conversely, the attenuated gut pro-inflammatory profile of CTRL and RF3 groups may reflect the reduced abundance of *Achromobacter* in combination with a host down-regulated expression of *il1b* and *tnfα.* Interestingly, *Achromobacter* and *tnfa* in AI were also positively correlated, reinforcing the possible host-microbe interaction at the immune system level.

Bacterial shifts in the SK microbiome highlighted the inferred over-representation of the pyridoxal 5’-phosphate (PLP) biosynthesis I in RF1 and RF2 fish in comparison to the clustered CTRL_RF3 group. PLP is the biologically active form of vitamin B6, with a key role in maintaining the structural and mechanical integrity of bone and muscle connective tissues [96]. Humans and most animals, including fish, lack the ability to synthesize this vitamin that must be obtained from the diet, relying on complex *de novo* biosynthetic pathways carried out by bacteria and fungi [97]. Indeed, in our study, several abundant SK bacteria taxa, including *Exiguobacterium* and *Planococcus*, would be able to synthesize or utilizing PLP-dependent enzymes [98–100]. Such metabolic feature may be connected with the up-regulated muscle regeneration pathways, highlighted by the increased expression of *myod2*, *mrf4*, and *cpst* genes in RF fish [23]. In any case, this potential connection between host and SK microbiome is inferred from metagenomic analyses and it requires further experimental validation.

Lastly, analysing at a closer look the WATER microbiome, it must be noted that the presence of fish already induced marked effects on microbial communities, increasing evenness under absence or low doses of RFs, while higher RF exposures reversed this pattern, bringing evenness back to IW. This outcome aligns with the recognized role of fish as ecosystem engineers, since they contribute with organic matter in the form of excretions, mucus, and sloughed epithelial cells, which act as substrates stimulating microbial proliferation and diversity [101]. By contrast, microplastics are well known to provide selective artificial surfaces that promote colonization by specific microbial taxa at the expense of others, ultimately reducing overall microbial diversity [102]. These opposite influences highlight the complex interplay between natural biological drivers and anthropogenic pollutants in shaping aquatic microbiota. Beyond these general diversity shifts, the WATER microbiome stood out as the most RF responsive compartment, both in terms of structure and function.

Compared to host-associated microbiomes, the WATER community displayed a strikingly higher number of discriminant taxa (691) and over-represented microbial functions (28). This pronounced sensitivity may be partly explained by its lower inter-replicate variability, which makes narrower fluctuations statistically detectable, but also by its direct exposure to both fish-derived inputs and dietary RFs. Importantly, these microbial shifts occurred in a flow-through system with complete tank water renewal every 2–3 h, a feature that would normally dilute transient microbial signals. The fact that distinct and diet-dependent alterations were still detected highlights the strength and persistence of the selective pressures generated by fish activity and RF exposure, which were robust enough to override the homogenizing effect of rapid water turnover. This finding underscores that fish presence and feeding regime, not husbandry conditions, can be the primary drivers of microbial community dynamics, as similarly observed in rainbow trout fed *Tenebrio molitor*–based diets [103]. Moreover, the capacity of RFs to reshape the microbiota even under constant water tank refreshment reinforces their potential ecological relevance in aquaculture environments.

Otherwise, the identity of the dominant WATER taxa provides further insights into how RF exposure reshaped the aquatic microbiota in the presence of fish. In that sense, it must be noted that the WATER microbiome was primarily dominated by *Synechococcu*s, a globally distributed Cyanobacteriota genus fundamental to aquatic ecosystem functioning [104]. *Synechococcus* plays an essential role in primary production, biogeochemical cycling, and CO₂ fixation [105, 106], being particularly important in the Mediterranean Sea [107]. Thus, the observed decline in *Synechococcus* populations, particularly under higher diet RF loads, raises concerns about the potential impacts of RFs on the natural ecosystem productivity, nutrient cycling, and overall resilience. In an ecological aquaculture context, a reduction in *Synechococcus* may have far ecosystem consequences. As a key primary producer, its decline can diminish oxygen production and carbon fixation, weakening the trophic base of the rearing system. Moreover, disruptions in nitrogen and phosphorus cycling may impair water quality and alter the balance between autotrophic and heterotrophic microbes. Such shifts often favour opportunistic or potentially pathogenic taxa, decreasing system stability and increasing the vulnerability of fish to environmental stress or disease [108]. Furthermore, the decline in *Synechococcus* was not an isolated event but coincided with broader shifts in other microbial groups. Thus, the over-representation of Gracilibacteria taxa paralleled the decline of both of *Synechococcus* genus and *Flavobacteriaceae* family. Members of the *Flavobacteriaceae* family are widely regarded as hotspots for MP biodegradation and nitrogen cycling [109]. However, both in this and previous studies using polyethylene, polystyrene, polycaprolactone and polylactic acid polymers, it has been reported a reduced abundance under cellulosic microfibres exposure [110]. Conversely, the rise of Gracilibacteria, which even surpassed *Synechococcus* abundance in RF2_RF3 fish, suggests a functional redirection of the WATER microbiome. Indeed, Gracilibacteria taxa has been associated with biofilms on medical masks submerged in coastal waters polluted by artisanal fisheries and hydrocarbons [111]. Indeed, genomic analyses indicate that members of this class harbour oxidases and hydrolases capable of degrading complex organic compounds, facilitating quorum sensing interactions, and contributing to cellulosic microfibre breakdown [112, 113]. In line with these findings, our inferred metagenomic analysis showed that RF exposure modulated the WATER microbiome towards a MP-degrading state. Over-represented pathways included those related to hydrocarbon degradation (limonene, pinene, ethylbenzene) as well as starch and sucrose metabolism. This metabolic reprogramming, together with the taxonomic shifts described above, suggests that dietary RFs not only reshape microbial composition but also rewire community-level functions when fish are part of the process. However, further validation of the actual metabolic activity must be undergone to ensure the real effects of bacterial community on hydrocarbon rewire. Altogether, our findings point to a dual ecological consequence: on one hand, the loss of key photosynthetic taxa like *Synechococcus* threatens ecosystem productivity; on the other, the rise of degradative consortia like Gracilibacteria may enhance microfibres breakdown. Notably, these dynamics unfold within the broader context of an emerging rearing water plastisphere, where plastics serve as novel microbial habitats with potential to alter host–environment interactions. The balance between these processes will be crucial to determine whether aquatic ecosystems under microfibre exposure move towards functional resilience or ecological instability.

## Conclusions

**Fig. 11:**
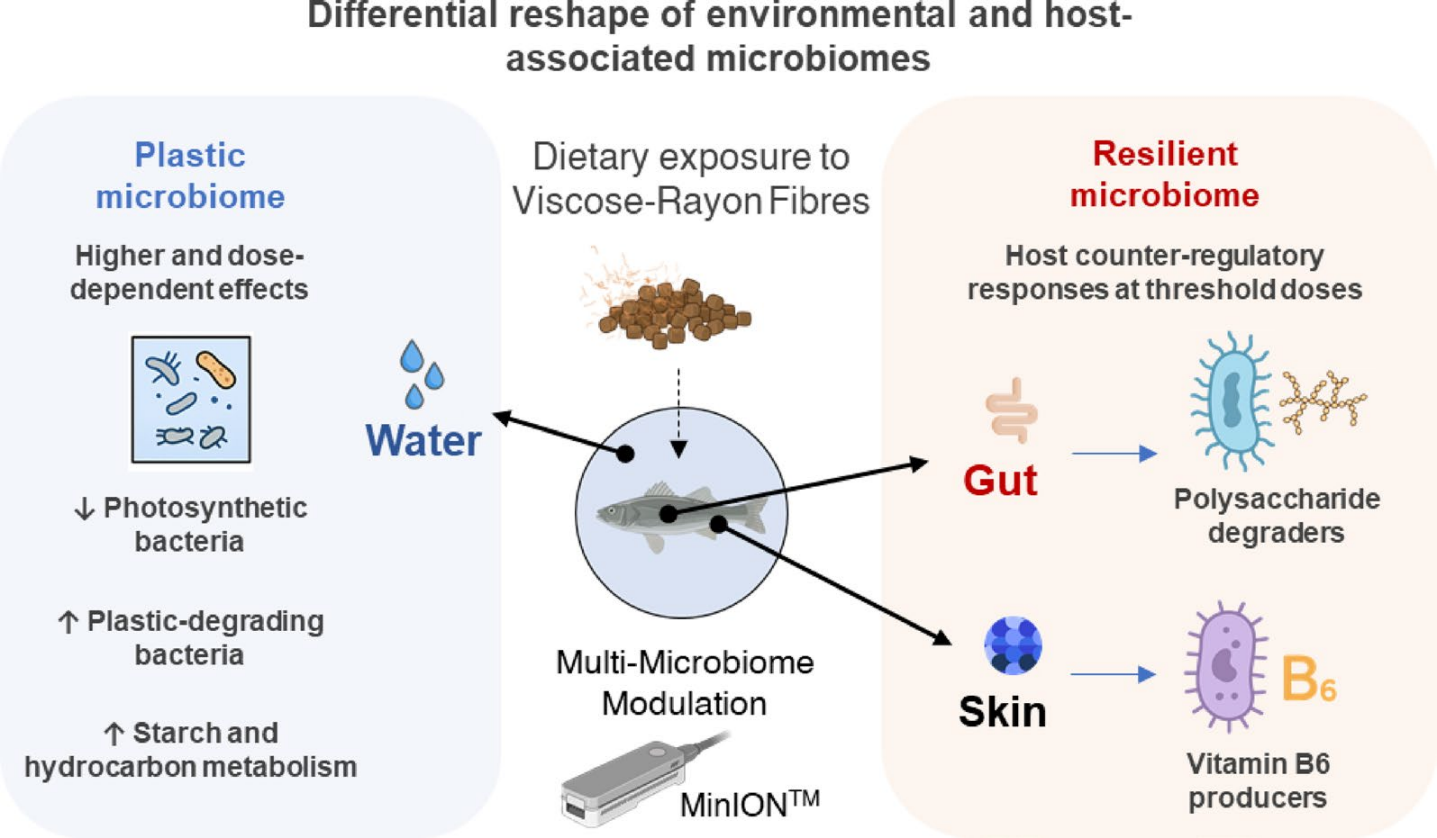
Schematic representation of the proposed model for integrative microbiome responses of European sea bass exposed to increasing concentrations of dietary RFs. Pathways included in this figure are inferred, and only reflects the metabolic potential of the bacterial communities associated to the RFs

This integrative study reveals, for the first time, that dietary cellulosic microfibres elicit compartment-specific microbiome responses in European sea bass aquaculture, with the rearing water microbiome being the most sensitive, the skin microbiome showing dose-dependent convergence toward the water community, and the gut microbiome displaying notable resilience. Across compartments, we identify a dose-dependent response, but also a concomitant dose-threshold pattern, whereby moderate RF exposure disrupts diversity and community structure, but the highest dose triggers a partial return toward control-like configurations. These findings highlight that RFs can shape host-associated and environmental microbiota even under rapid water-exchange conditions, underscoring the strength of fish-driven and feed-derived microbial pressures. We also identified key microbial taxa and pathways involved in RF degradation, vitamin biosynthesis, inflammation, and nutrient cycling, as well as critical host–microbiome interactions (Fig. 11). For sustainable aquaculture, our work indicates that RF-based feeds may be less disruptive than synthetic microplastics, but RF levels must be carefully regulated, as intermediate doses may pose higher microbiome sensitivity than expected. Future studies should prioritize validating these predicted microbial functions to fully unravel the molecular crosstalk between host and microbiota under microfibres-driven pollution stress. It will also be crucial to evaluate the temporal persistence of RF-induced alterations in microbiota and host responses, in order to determine whether such changes are transient or sustained over time. Overcoming these limitations, the definition of the rearing water plastisphere can be closer, together with its validation within aquaculture settings. This could ultimately facilitate the development of effective, minimally invasive methods for the early detection of microplastic-induced physiological and environmental disturbances in aquaculture production systems.

## Supplementary Information

Supplementary Material 1: Supplementary Table 1. Table showing the detailed sequencing data obtained in this study. Supplementary Table 2. Taxa with minimum VIP values responsible of the separation of samples by diet in the different groups of RFs doses. VIP values represent the variable importance in projection after component 1. Supplementary Table 3. List of AI potential stable and dose-insensitive functions detected in the study. Supplementary Table 4. List of significant (p < 0.05, |CC| > 0.6) correlations between discriminant taxa and discriminant genes retrieved from Matias et al. [23]. Supplementary Figure 1. Rarefaction curves obtained from the sequencing data of the 87 samples included in this study. Supplementary Figure 2. Non-metric multidimensional scaling (NMDS) ordination plot based on Bray–Curtis dissimilarity of relative microbial abundances in (A) CTRL, (B) RF1, (C) RF2 and (D) RF3, illustrating the community structure among gut, skin, and water microbiomes. Stress values were < 0.2, indicating a reliable two-dimensional representation. Ellipses represent 95% confidence intervals calculated using Mahalanobis distances. Centroids denote the average community position for each microbiome type. Supplementary Figure 3. Principal Component Analysis (PCA) of relative microbial abundances in (A) AI, (B) SK and (C) WATER microbiomes. The first two principal components are shown, explaining 20-44% of the total variance. Each point represents a sample with closer values indicating stronger similarity in multivariate profiles.

## Data Availability

Raw sequencing data are available at NCBI’s Sequence Read Archive under accession PRJNA1282057 (BioSample accession numbers: SAMN49609176-262).
