# Supplementary material for: Dietary rayon microfibers differentially reshape rearing water and host associated microbiomes of farmed European sea bass (Dicentrarchus labrax)
Source: Environ Microbiome. 2026 Jan 17;21:30. doi: 10.1186/s40793-026-00851-5 (PMC12896088; doi:10.1186/s40793-026-00851-5)
Supplement: Supplementary file 1 — Supplementary Material 1: Supplementary Table 1. Table showing the detailed sequencing data obtained in this study. Supplementary Table 2. Taxa with minimum VIP values responsible of the separation of samples by diet in the different groups of RFs doses. VIP values represent the variable importance in projection after component 1. Supplementary Table 3. List of AI potential stable and dose-insensitive functions detected in the study. Supplementary Table 4. List of significant (p < 0.05, |CC| > 0.6) correlations between discriminant taxa and discriminant genes retrieved from Matias et al. [23]. Supplementary Figure 1. Rarefaction curves obtained from the sequencing data of the 87 samples included in this study. Supplementary Figure 2. Non-metric multidimensional scaling (NMDS) ordination plot based on Bray–Curtis dissimilarity of relative microbial abundances in (A) CTRL, (B) RF1, (C) RF2 and (D) RF3, illustrating the community structure among gut, skin, and water microbiomes. Stress values were < 0.2, indicating a reliable two-dimensional representation. Ellipses represent 95% confidence intervals calculated using Mahalanobis distances. Centroids denote the average community position for each microbiome type. Supplementary Figure 3. Principal Component Analysis (PCA) of relative microbial abundances in (A) AI, (B) SK and (C) WATER microbiomes. The first two principal components are shown, explaining 20-44% of the total variance. Each point represents a sample with closer values indicating stronger similarity in multivariate profiles. [file 40793_2026_851_MOESM1_ESM.zip › Supplementary Table 1. Sequencing Metrics.docx]

| **Supplementary Table 1**. Sequencing Metrics of the 87 samples in this study. | | | | | | | | | | |
| --- | --- | --- | --- | --- | --- | --- | --- | --- | --- | --- |
| **AI-Sample** | **Raw** | **Assigned** |  | **SK-Sample** | **Raw** | **Assigned** |  | **WATER-Sample** | **Raw** | **Assigned** |
| AI-CTRL_1 | 64876 | 53177 |  | SK-CTRL_1 | 68819 | 56409 |  | WATER-CTRL_1 | 439505 | 360250 |
| AI-CTRL_2 | 33748 | 27662 |  | SK-CTRL_2 | 123746 | 101431 |  | WATER-CTRL_2 | 213230 | 174779 |
| AI-CTRL_3 | 25877 | 21211 |  | SK-CTRL_3 | 60373 | 49486 |  | WATER-CTRL_3 | 308810 | 253123 |
| AI-CTRL_4 | 78181 | 64083 |  | SK-CTRL_4 | 99007 | 81153 |  | WATER-RF1_1 | 175870 | 144156 |
| AI-CTRL_5 | 56834 | 46585 |  | SK-CTRL_5 | 90839 | 74458 |  | WATER-RF1_2 | 153946 | 126185 |
| AI-CTRL_6 | 41386 | 33923 |  | SK-CTRL_6 | 110548 | 90613 |  | WATER-RF1_3 | 171229 | 140352 |
| AI-CTRL_7 | 39943 | 32740 |  | SK-CTRL_7 | 95073 | 77929 |  | WATER-RF2_1 | 144123 | 118134 |
| AI-CTRL_8 | 119539 | 97983 |  | SK-CTRL_8 | 71795 | 58848 |  | WATER-RF2_2 | 146784 | 120315 |
| AI-CTRL_9 | 181621 | 148870 |  | SK-CTRL_9 | 115090 | 94336 |  | WATER-RF2_3 | 153914 | 126159 |
| AI-RF1_1 | 13385 | 10971 |  | SK-RF1_1 | 30534 | 25028 |  | WATER-RF3_1 | 276530 | 226664 |
| AI-RF1_2 | 45449 | 37253 |  | SK-RF1_2 | 61672 | 50551 |  | WATER-RF3_2 | 306111 | 250911 |
| AI-RF1_3 | 11800 | 9672 |  | SK-RF1_3 | 15099 | 12376 |  | WATER-RF3_3 | 59770 | 48992 |
| AI-RF1_4 | 15171 | 12435 |  | SK-RF1_4 | 22626 | 18546 |  | IW_1 | 325855 | 267094 |
| AI-RF1_5 | 22555 | 18488 |  | SK-RF1_5 | 33585 | 27529 |  | IW_2 | 262313 | 215011 |
| AI-RF1_6 | 9450 | 7746 |  | SK-RF1_6 | 67482 | 55313 |  | IW_3 | 377696 | 309587 |
| AI-RF1_7 | 8860 | 7262 |  | SK-RF1_7 | 72605 | 59512 |  |  |  |  |
| AI-RF1_8 | 6276 | 5144 |  | SK-RF1_8 | 59997 | 49178 |  |  |  |  |
| AI-RF1_9 | 9289 | 7614 |  | SK-RF1_9 | 22975 | 18832 |  |  |  |  |
| AI-RF2_1 | 112602 | 92297 |  | SK-RF2_1 | 42885 | 35152 |  |  |  |  |
| AI-RF2_2 | 23844 | 19544 |  | SK-RF2_2 | 53219 | 43622 |  |  |  |  |
| AI-RF2_3 | 9529 | 7811 |  | SK-RF2_3 | 9883 | 8101 |  |  |  |  |
| AI-RF2_4 | 18137 | 14866 |  | SK-RF2_4 | 52276 | 42849 |  |  |  |  |
| AI-RF2_5 | 15259 | 12507 |  | SK-RF2_5 | 34543 | 28314 |  |  |  |  |
| AI-RF2_6 | 12218 | 10015 |  | SK-RF2_6 | 43999 | 36065 |  |  |  |  |
| AI-RF2_7 | 24682 | 20231 |  | SK-RF2_7 | 77285 | 63348 |  |  |  |  |
| AI-RF2_8 | 77807 | 63776 |  | SK-RF2_8 | 50596 | 41472 |  |  |  |  |
| AI-RF2_9 | 13400 | 10984 |  | SK-RF2_9 | 29146 | 23890 |  |  |  |  |
| AI-RF3_1 | 217877 | 178588 |  | SK-RF3_1 | 91047 | 74629 |  |  |  |  |
| AI-RF3_2 | 35561 | 29148 |  | SK-RF3_2 | 71287 | 58432 |  |  |  |  |
| AI-RF3_3 | 44988 | 36875 |  | SK-RF3_3 | 181215 | 148537 |  |  |  |  |
| AI-RF3_4 | 93395 | 76553 |  | SK-RF3_4 | 9066 | 7431 |  |  |  |  |
| AI-RF3_5 | 60357 | 49473 |  | SK-RF3_5 | 151875 | 124488 |  |  |  |  |
| AI-RF3_6 | 35195 | 28848 |  | SK-RF3_6 | 153355 | 125701 |  |  |  |  |
| AI-RF3_7 | 5048 | 4138 |  | SK-RF3_7 | 147794 | 121143 |  |  |  |  |
| AI-RF3_8 | 34485 | 28266 |  | SK-RF3_8 | 48041 | 39378 |  |  |  |  |
| AI-RF3_9 | 164083 | 134494 |  | SK-RF3_9 | 100415 | 82307 |  |  |  |  |
