# Supplementary material for: Dietary rayon microfibers differentially reshape rearing water and host associated microbiomes of farmed European sea bass (Dicentrarchus labrax)
Source: Environ Microbiome. 2026 Jan 17;21:30. doi: 10.1186/s40793-026-00851-5 (PMC12896088; doi:10.1186/s40793-026-00851-5)
Supplement: Supplementary file 1 — Supplementary Material 1: Supplementary Table 1. Table showing the detailed sequencing data obtained in this study. Supplementary Table 2. Taxa with minimum VIP values responsible of the separation of samples by diet in the different groups of RFs doses. VIP values represent the variable importance in projection after component 1. Supplementary Table 3. List of AI potential stable and dose-insensitive functions detected in the study. Supplementary Table 4. List of significant (p < 0.05, |CC| > 0.6) correlations between discriminant taxa and discriminant genes retrieved from Matias et al. [23]. Supplementary Figure 1. Rarefaction curves obtained from the sequencing data of the 87 samples included in this study. Supplementary Figure 2. Non-metric multidimensional scaling (NMDS) ordination plot based on Bray–Curtis dissimilarity of relative microbial abundances in (A) CTRL, (B) RF1, (C) RF2 and (D) RF3, illustrating the community structure among gut, skin, and water microbiomes. Stress values were < 0.2, indicating a reliable two-dimensional representation. Ellipses represent 95% confidence intervals calculated using Mahalanobis distances. Centroids denote the average community position for each microbiome type. Supplementary Figure 3. Principal Component Analysis (PCA) of relative microbial abundances in (A) AI, (B) SK and (C) WATER microbiomes. The first two principal components are shown, explaining 20-44% of the total variance. Each point represents a sample with closer values indicating stronger similarity in multivariate profiles. [file 40793_2026_851_MOESM1_ESM.zip › Supplementary Table 3. Stable and dose-insensitive functions in AI.docx]

| **Supplementary Table 3**. List of AI potential stable and dose-insensitive functions detected in the study. | | | |
| --- | --- | --- | --- |
| **Pathway** | **Description** | **LVL2** | **LVL1** |
| ko00071 | Fatty acid metabolism | Global and overview maps | Metabolism |
| ko00660 | C5-Branched dibasic acid metabolism | Carbohydrate metabolism | Metabolism |
| ko00620 | Pyruvate metabolism | Carbohydrate metabolism | Metabolism |
| ko00020 | Citrate cycle (TCA cycle) | Carbohydrate metabolism | Metabolism |
| ko00030 | Pentose phosphate pathway | Carbohydrate metabolism | Metabolism |
| ko00650 | Butanoate metabolism | Carbohydrate metabolism | Metabolism |
| ko00010 | Glycolysis / Gluconeogenesis | Carbohydrate metabolism | Metabolism |
| ko00640 | Propanoate metabolism | Carbohydrate metabolism | Metabolism |
| ko00630 | Glyoxylate and dicarboxylate metabolism | Carbohydrate metabolism | Metabolism |
| ko00521 | Streptomycin biosynthesis | Biosynthesis of other secondary metabolites | Metabolism |
| ko00983 | Drug metabolism - other enzymes | Xenobiotics biodegradation and metabolism | Metabolism |
| ko00061 | Fatty acid biosynthesis | Lipid metabolism | Metabolism |
| ko00290 | Valine, leucine and isoleucine biosynthesis | Amino acid metabolism | Metabolism |
| ko00250 | Alanine, aspartate and glutamate metabolism | Amino acid metabolism | Metabolism |
| ko00260 | Glycine, serine and threonine metabolism | Amino acid metabolism | Metabolism |
| ko00280 | Valine, leucine and isoleucine degradation | Amino acid metabolism | Metabolism |
| ko00300 | Lysine biosynthesis | Amino acid metabolism | Metabolism |
| ko00340 | Histidine metabolism | Amino acid metabolism | Metabolism |
| ko00480 | Glutathione metabolism | Metabolism of other amino acids | Metabolism |
| ko00450 | Selenocompound metabolism | Metabolism of other amino acids | Metabolism |
| ko00550 | Peptidoglycan biosynthesis | Glycan biosynthesis and metabolism | Metabolism |
| ko00540 | Lipopolysaccharide biosynthesis | Glycan biosynthesis and metabolism | Metabolism |
| ko00770 | Pantothenate and CoA biosynthesis | Metabolism of cofactors and vitamins | Metabolism |
| ko00670 | One carbon pool by folate | Metabolism of cofactors and vitamins | Metabolism |
| ko00785 | Lipoic acid metabolism | Metabolism of cofactors and vitamins | Metabolism |
| ko00760 | Nicotinate and nicotinamide metabolism | Metabolism of cofactors and vitamins | Metabolism |
| ko00750 | Vitamin B6 metabolism | Metabolism of cofactors and vitamins | Metabolism |
| ko00780 | Biotin metabolism | Metabolism of cofactors and vitamins | Metabolism |
| ko00790 | Folate biosynthesis | Metabolism of cofactors and vitamins | Metabolism |
| ko00730 | Thiamine metabolism | Metabolism of cofactors and vitamins | Metabolism |
| ko01051 | Biosynthesis of ansamycins | Metabolism of terpenoids and polyketides | Metabolism |
| ko01055 | Biosynthesis of vancomycin group antibiotics | Metabolism of terpenoids and polyketides | Metabolism |
| ko00970 | Aminoacyl-tRNA biosynthesis | Translation | Genetic Information Processing |
| ko04122 | Sulfur relay system | Folding, sorting and degradation | Genetic Information Processing |
| ko03060 | Protein export | Folding, sorting and degradation | Genetic Information Processing |
| ko03430 | Mismatch repair | Replication and repair | Genetic Information Processing |
| ko03440 | Homologous recombination | Replication and repair | Genetic Information Processing |
| ko02010 | ABC transporters | Membrane transport | Environmental Information Processing |
| ko04112 | Cell cycle - Caulobacter | Cell growth and death | Cellular Processes |
| ko02030 | Bacterial chemotaxis | Cell motility | Cellular Processes |
| ko02040 | Flagellar assembly | Cell motility | Cellular Processes |
